# Distributions of the HLA-A, HLA-B, HLA-C, HLA-DRB1, and HLA-DQB1 alleles and haplotype frequencies of 1763 stem cell donors in the Colombian Bone Marrow Registry typed by next-generation sequencing

**DOI:** 10.3389/fimmu.2022.1057657

**Published:** 2023-01-09

**Authors:** David G. Hernández-Mejía, Iván Aurelio Páez-Gutiérrez, Valerie Dorsant Ardón, Nathalie Camacho Ramírez, Melissa Mosquera, Paola Andrea Cendales, Bernardo Armando Camacho

**Affiliations:** Instituto Distrital de Ciencia, Biotecnología e Innovación en Salud - IDCBIS, Bogotá, Colombia

**Keywords:** allele frequency, Bone Marrow Registry, Colombia, HLA, next-generation sequencing

## Abstract

The HLA compatibility continues to be the main limitation when finding compatible donors, especially if an identical match is not found within the patient’s family group. The creation of bone marrow registries allowed a therapeutic option by identifying 10/10 compatible unrelated donors (URD). However, the availability and frequency of haplotypes and HLA alleles are different among ethnic groups and geographical areas, increasing the difficulty of finding identical matches in international registries. In this study, the HLA-A, -B, -C, -DRB1, and -DQB1 loci of 1763 donors registered in the Colombian Bone Marrow Registry were typed by next-generation sequencing. A total of 52 HLA-A, 111 HLA-B, 41 HLA-C, 47 HLA-DRB1, and 20 HLA-DQB1 alleles were identified. The 3 most frequent alleles for each loci were A*24:02g (20,8%), A*02:01g (16,1%), A*01:01g (7.06%); B*35:43g (7.69%), B*40:02g (7.18%), B*44:03g (6.07%); C*04:01g (15.40%), C*01:02g (10.49%), C*07:02g (10.44%); DRB1*04:07g (11.03%), DRB1*07:01g (9.78%), DRB1*08:02g (6.72%); DQB1*03:02g (20.96%), DQB1*03:01g (17.78%) and DQB1*02:01g (16.05%). A total of 497 HLA-A-C-B-DRB1-DQB1 haplotypes were observed with a frequency greater than or equal to 0.05% (> 0.05%); the haplotypes with the highest frequency were A*24:02g~B*35:43g~C*01:02g~DQB1*03:02g~DRB1*04:07g (3.34%), A*29:02g~B*44:03g~C*16:01g~DQB1*02:01g~DRB1*07:01g (2.04%), and A*01:01g~B*08:01g~C*07:01g~DQB1*02:01g~DRB1*03:01g (1.83%). This data will allow the new Colombian Bone Marrow Donor Registry to assess the genetic heterogeneity of the Colombian population and serve as a tool of interest for future searches of unrelated donors in the country.

## 1 Introduction

Hematopoietic stem cell transplantation continues to be one of the most widely used treatments for both malignant and benign hematological diseases (1–4). In recent decades, unrelated donors (URD) have been the first option for transplantation when an HLA-compatible family donor is not available. In countries with a National Bone Marrow (BM) Donors Registry, finding an 8/8 or 10/10 compatible URD can be reduced by several months, having a positive effect on the treatment and quality of life of patients (5–13). In the absence of a National BM Donors Registry, searching for compatible URD can take a longer time, leading medical treatment toward alternative sources of hematopoietic stem cells such as haploidentical transplant or umbilical cord blood (UCB) units, which are associated with higher risks of graft-versus-host disease (GVHD), graft failure or transplant-related death (TRM) (14–18). Since 2018, the *Instituto Distrital de Ciencia, Biotecnología e Innovación en Salud* (IDCBIS) has been promoting the creation of a National BM Donor Registry in Colombia to provide hematopoietic stem cells from URD to the country’s transplant centers, ultimately shortening the waiting time of patients requiring HSC transplants. Currently, 4435 donors have registered in the Colombia BM Donor Registry, and, by the time of this study 1779 were HLA typed.

The major histocompatibility complex (MHC), known in humans as the human leukocyte antigen (HLA) system, includes proteins responsible for mediating antigen presentation to CD8+ and CD4+ lymphocytes and thus activating the cellular and humoral immune responses in individuals. These genes are contained in approximately 4 MB located on the short arm of chromosome 6 (6p21). The MHC is composed of 3 subregions: HLA classes I, II, and III. Within class I are classical (HLA-A, -B and -C) and nonclassical (HLA-E, -F, -G and HFE) HLA genes. Likewise, within class II, classic genes such as HLA-DRB1, -DRB3/4/5, -DQB1, and -DPB1, and nonclassical genes (HLA-DM and HLA-DO). In between the HLA class III region is found, where there are genes participating in the immune response, such as complement System proteins and cytokines; however, class III genes differ in their structure and function compared to the genes class I and II. The HLA system has the highest density of genes in the human genome which are highly polymorphic. HLA alleles and haplotype frequencies vary widely between different populations, and even in the same territory may be conditioned by ethnicities and geographical regions (19–24).

Access to next-generation sequencing (NGS) technologies allowed the identification of a vast number of new HLA alleles, thus expanding the records in the IPD-IMGT/HLA database (25, 26), 36016 HLA alleles are recorded in the latest version (V 3.50) of the database released in October 2022, 25019 HLA class I alleles, and 10201 HLA class II alleles. HLA compatibility between the recipient and the donor is currently the main criterion for donor selection since numerous reports documented that a disparity in any of these genes leads to a worse transplant outcome and an increase in GVHD (15, 16, 27–33).

Colombia is in the extreme northeast of the South American continent, bordered by Panamá to the northeast, Perú and Ecuador to the south, Venezuela to the west, and Brazil to the southwest, in addition to having access to the Pacific Ocean on the eastern side and the Caribbean Sea to the north. This exceptional geographical position made Colombia a necessary passage between the pre-Columbian cultures that migrated from the south to the north of the American continent and vice versa (34, 35). Due to the colonization process in the fifteenth century by the Spanish empire, the first European population settlement occurred, as well as the first inhabitants of African origin arrived in the territory as a result of the slave trade. These Afro-American populations ended up settling mainly on the Pacific and Caribbean coasts of the country. In the territory, processes of miscegenation occurred over several generations between the European Caucasian, Native American, and African American populations, giving way to mestizo (Caucasian with Native American), mulatto (Caucasian with African American), and Zambo (Native American with African American) populations (36, 37).

Since the independence of the country in the 19th century, there have been continuous displacements of the population from rural areas to the urban centers of the country as a result of different civil wars, political violence in the mid-20th century, and in recent decades, the conflict between different armed groups (guerrillas, paramilitaries, and drug traffickers) (38, 39). Noteworthy during the 20th century occurs the migration of Turkish-Lebanese people who settled in the commercial ports of the Atlantic coast (40). The current population of Colombia is 50,372,424, concentrated in urban centers of the Andean region and the Caribbean coast, with the most populated cities being Bogotá (7,743,955 people), Medellín (2,533,424 people), Cali (2,252,616 people) and Barranquilla (1,274,250). Regarding their ethnic composition, 88.9% of the population is mestizo or non-Native American, 6.7% are Afro-descendant, 4.31% are Native American, 0.06% are Raizal, and 0.01% are Roma-Gypsy (41, 42).

This complex sociocultural, geographical and historical context in Colombia has produced a gene pool that currently has not been fully characterized. The objective of this study is to describe the allele frequencies and haplotypes of the classic HLA genes, using high-resolution NGS techniques, from donors registered in the Colombian BM Donor Registry; the information obtained will allow a better understanding of the distribution of HLA genes in different geographical regions of the country. Furthermore, the data obtained will be a resource for the national BM donor registry in Colombia to establish donor recruitment strategies.

## 2 Materials and methods

### 2.1 Population sample

**Figure 1:**
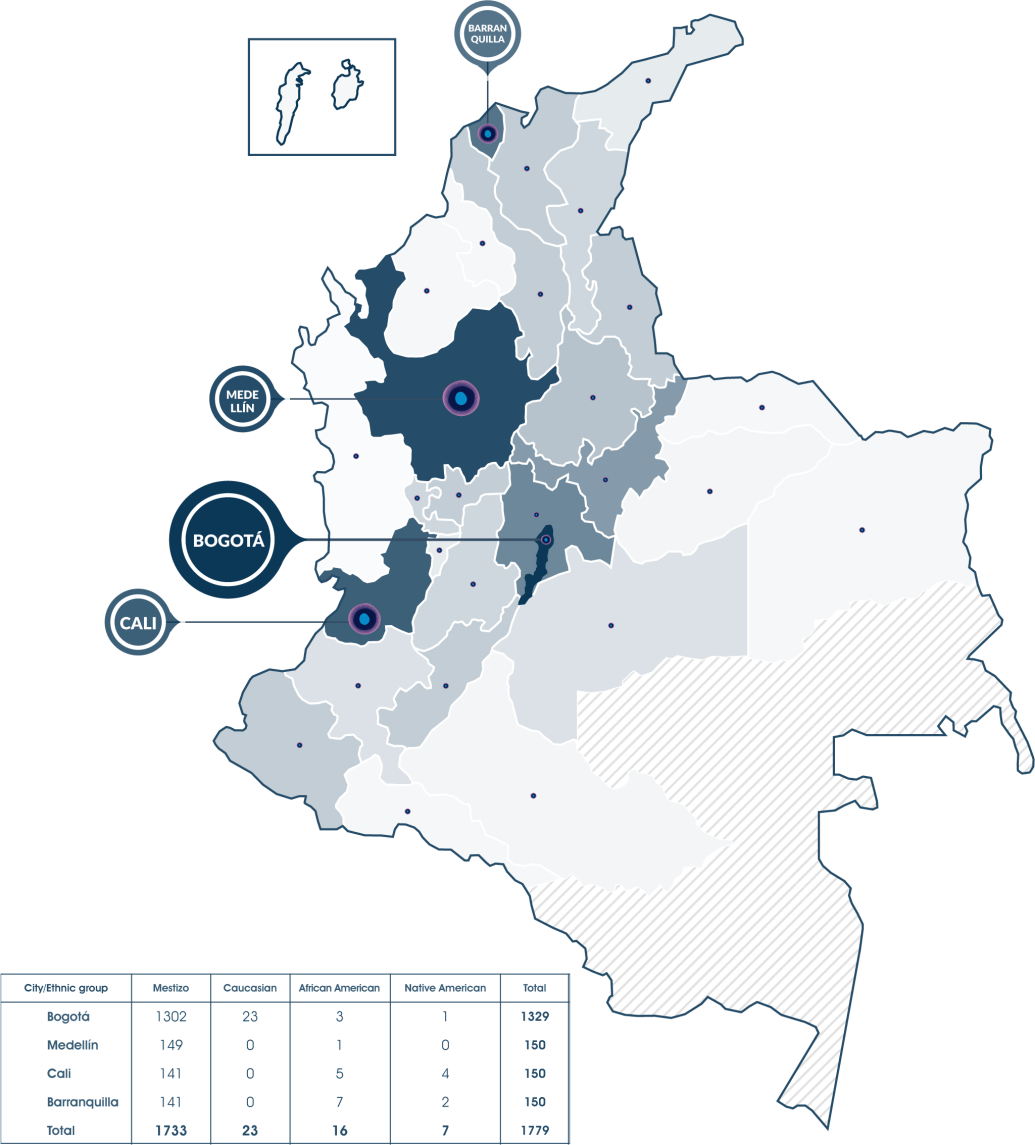
1779 donors were registered in the Colombian Bone Marrow Donor Registry, 1749 of them are Colombian and 30 are foreigners residing in the country, they all were recruited in 4 major cities: Bogotá, Cali, Medellín and Barranquilla. Currently, people from Bogotá and 28 of the 32 Colombian departments (except Amazonas, Vaupés, Guaviare, and Guainía) had registered.

This study included 1779 samples from donors enrolled from 2018 to 2021 in the Colombian BM donor registry. All donors signed an informed consent form approved by the Ethics Committee of the *Secretaría Distrital de Salud de Bogotá*. Additionally, each donor completed a form containing demographic data such as place of birth, ethnicity, age, and gender. The registration process was carried out in 4 of the largest cities of the country: 1329 in Bogotá, 150 in Medellín, 150 in Cali, and 150 in Barranquilla. 1749 of the recruited donors, were Colombian nationals (**Figure 1**; **Supplementary Table 1**), while the other 30 were foreigners (**Supplementary Figure 1**). Donors self-identify as mestizo (97.4%), Caucasian (1.3%), African American (0.9%), and Native American (0.4%). For the genotyping of the HLA-A, HLA-B, HLA-C, HLA-DRB1, and HLA-DQB1 loci, samples of blood stored in Protein Saver^®^ cards, or buccal swabs were taken from 1347 women and 416 men, for a total of 1763 registries analyzed. 16 results were pending, 3 of them for confirmation of possible new HLA alleles. These 16 results were not included in the statistical analysis.

### 2.2 HLA typing

Donors were typed at high resolution for the HLA-A, HLA-B, HLA-C, HLA-DRB1, and HLA-DQB1 loci. The blood samples were sent for sequencing to Histogenetics (Ossining, NY), and the buccal cell swabs were sent to DKMS Life Science Lab (Dresden, Germany). Both laboratories used Illumina platforms for sequencing exons 2 and 3 of HLA class I loci, and exon 2 of HLA class II loci, following the protocols standardized by each laboratory (43, 44). Typing results for each HLA allele were coded by G groups, with at least the first 2 fields being always resolved. New alleles identified at typing were confirmed and submitted by each laboratory to the IMGT/HLA database. Reports of these new alleles were subsequently published by the Colombian BM Donor Registry.

To corroborate the homozygous typing results, these labs use multiple sequencing technologies and platforms during the HLA testing. Using a combination of generic and group-specific primers complementary to sequences in the exon 2 and 3 and intron 2 for HLA class I genes and the exon 2 for HLA class II can generate amplicons to confirm the homozygosity. In case of doubt, the largest amplicons are repeated with PacBio sequencing technology. In addition, known associations between the different genes are checked before to reaffirm their homozygous associations.

### 2.3 Statistical analysis

HLA typing results were coded by g-groups for statistical analysis. The allele frequencies (AFs) of HLA-A, HLA-B, HLA-C, HLA-DRB1, and HLA-DQB1 loci were calculated by direct counting using Arlequin 3.5.2.2 (45), and haplotype frequencies (HFs) were calculated using the expectation-maximization (EM) algorithm from the open-source software Hapl-o-Mat (46). The haplotypes with a frequency lower than 10^-6^ were not reported. Hardy-Weinberg equilibrium (HWE) was determined for each locus using Fisher’s exact test and, linkage disequilibrium (LD) between allele pairs was also estimated using Arlequin 3.5.2.2 (45). A p-value of < 0.05 was considered significant.

## 3 Results

### 3.1 Allele frequency

In total 52 HLA-A alleles, 111 HLA-B alleles, 41 HLA-C alleles, 47 HLA-DRB1 alleles, and 20 HLA-DQB1 alleles were identified for the 1763 samples analyzed. Complete AFs results are provided in **Supplementary Table 2**.

The 20 most frequent alleles for HLA class I loci are shown in **Table 1**. For the HLA-A locus, 21 alleles had a frequency higher than 1%, and the most frequent alleles in this locus were A*24:02g (20.87%), A*02:01g (16.11%), A*01:01g (7.06%), A*03:01g (6.83%), and A*29:02g (5.22%). For the HLA-B locus, 27 alleles occurred at a frequency higher than 1%, and the most frequent alleles were B*35:43g (7.69%), B*40:02g (7.18%), B*44:03g (6.07%), B*51:01g (5.90%), and B*07:02g (5.36%). Finally, for HLA-C locus, 17 alleles had a frequency greater than 1%, being the most frequent alleles C*04:01g (15.40%), C*01:02g (10.49%), C*07:02g (10.44%), C*07:01g (8.93%), and C*03:04g (8.05%).

**Table 1:** List of the 20 most frequent alleles for HLA class I loci for the population in the study and a comparison with allele frequencies reported by the Colombian UCB Bank.

Position	HLA-A	Frequency	Frequency CBB Colombia	HLA-B	Frequency	Frequency CBB Colombia	HLA-C	Frequency	Frequency CBB Colombia
*1*	A*24:02 g	0.208735	0.2081	B*35:43 g	0.076858	0.0865	C*04:01 g	0.153999	0.1490
*2*	A*02:01 g	0.161089	0.1613	B*40:02 g	0.071753	0.0844	C*01:02 g	0.104935	0.1138
*3*	A*01:01 g	0.070618	0.0608	B*44:03 g	0.060692	0.0560	C*07:02 g	0.104368	0.0967
*4*	A*03:01 g	0.068349	0.0612	B*51:01 g	0.058990	0.0564	C*07:01 g	0.089336	0.0892
*5*	A*29:02 g	0.052184	0.0455	B*07:02 g	0.053602	0.0499	C*03:04 g	0.080545	0.0817
*6*	A*68:01 g	0.049915	0.0519	B*35:01 g	0.052467	0.0451	C*08:02 g	0.060692	0.0492
*7*	A*11:01 g	0.043676	0.0427	B*14:02 g	0.051049	0.0400	C*16:01 g	0.053602	0.0513
*8*	A*23:01 g	0.032048	0.0328	B*18:01 g	0.038571	0.0345	C*06:02 g	0.049631	0.0513
*9*	A*31:01 g	0.031764	0.0396	B*08:01 g	0.035167	0.0318	C*05:01 g	0.047646	0.0506
*10*	A*26:01 g	0.031764	0.0205	B*44:02 g	0.030913	0.0390	C*12:03 g	0.040272	0.0455
*11*	A*02:22 g	0.026943	0.0321	B*38:01 g	0.025241	0.0229	C*15:02 g	0.039138	0.0424
*12*	A*33:01 g	0.021271	0.0167	B*39:05 g	0.024957	0.0287	C*02:02 g	0.038571	0.0376
*13*	A*68:02 g	0.019853	0.0243	B*49:01 g	0.024390	0.0280	C*03:05 g	0.031764	0.0383
*14*	A*32:01 g	0.019285	0.0239	B*35:12 g	0.023256	0.0284	C*03:03 g	0.019002	0.0178
*15*	A*30:02 g	0.019002	0.0243	B*15:01 g	0.018434	0.0181	C*17:01 g	0.016733	0.0154
*16*	A*02:13	0.017584	0.0198	B*57:01 g	0.017867	0.0130	C*08:01 g	0.013330	0.0126
*17*	A*24:03 g	0.016449	0.0147	B*53:01 g	0.017016	0.0195	C*14:02 g	0.012762	0.0092
*18*	A*02:05 g	0.014748	0.0167	B*27:05 g	0.016449	0.0106	C*12:02 g	0.007374	0.0085
*19*	A*30:01 g	0.012195	0.0178	B*50:01 g	0.016449	0.0188	C*16:02 g	0.005105	0.0068
*20*	A*24:14	0.011628	0.0133	B*48:01 g	0.015598	0.0147	C*15:05 g	0.004821	0.0058

**Figure 2:**
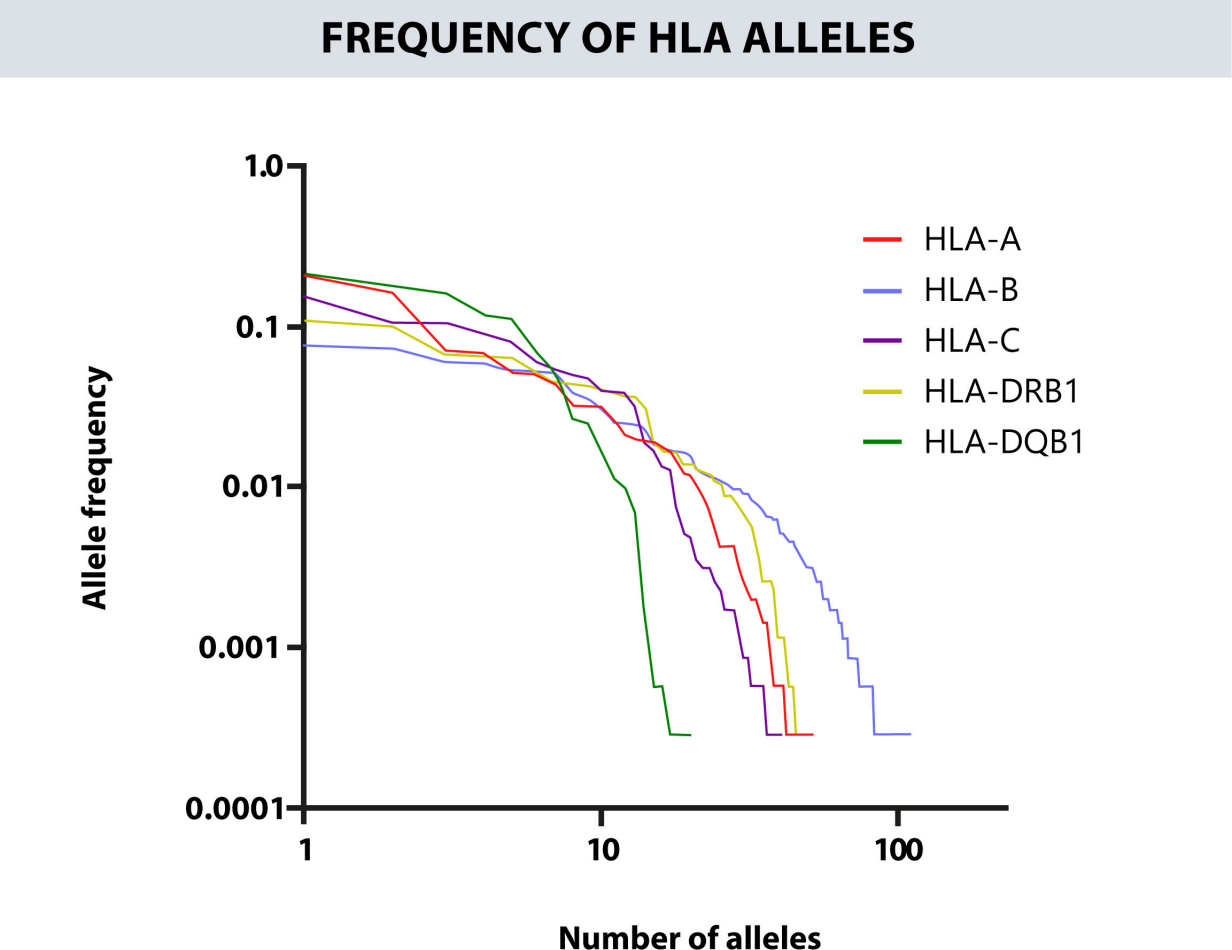
Represents the total number of alleles for each HLA loci versus its individual frequencies.

*Statistical significance (p < 0.05),

**Table 2** provides the 20 most frequent alleles for HLA class II loci. The most common HLA-DRB1 alleles were DRB1*04:07g (11.03%), DRB1*07:01g (9.78%), DRB1*08:02g (6.72%), DRB1*03:01g (6.58%), and DRB1*15:01g (6.38%). While the 5 most frequent alleles for HLA-DQB1 were DQB1*03:02g (20.96%), DQB1*03:01g (17.78%), DQB1*02:01g (16.05%), DQB1*05:01g (11.91%), and DQB1*04:02g (11.11%). Furthermore, 25 HLA-DRB1, and 11 HLA-DQB1 alleles were counted with frequencies greater than 1%. **Figure 2** shows the total number of alleles for each HLA loci and their individual frequency.

**Table 2:** List of the 20 most frequent alleles for HLA class II loci for the population in the study and a comparison with allele frequencies reported by the Colombian UCB Bank.

Position	HLA-DRB1	Frequency	Frequency CBB Colombia	HLA-DQB1	Frequency	Frequency CBB Colombia
*1*	DRB1*04:07 g	0.110323	0.1227	DQB1*03:02 g	0.209586	0.2245
*2*	DRB1*07:01 g	0.097845	0.0940	DQB1*03:01 g	0.177822	0.1907
*3*	DRB1*08:02 g	0.067215	0.0646	DQB1*02:01 g	0.160522	0.1459
*4*	DRB1*03:01 g	0.065797	0.0554	DQB1*05:01 g	0.119115	0.1169
*5*	DRB1*15:01 g	0.063812	0.0578	DQB1*04:02 g	0.111174	0.1087
*6*	DRB1*13:01 g	0.051333	0.0465	DQB1*06:02 g	0.070335	0.0707
*7*	DRB1*01:01 g	0.044526	0.0403	DQB1*06:03 g	0.051333	0.0458
*8*	DRB1*04:05 g	0.043392	0.0294	DQB1*06:04 g	0.026375	0.0236
*9*	DRB1*14:02 g	0.042258	0.0383	DQB1*03:03 g	0.024957	0.0222
*10*	DRB1*13:02 g	0.039705	0.0386	DQB1*05:03 g	0.017016	0.0174
*11*	DRB1*01:02 g	0.039138	0.0393	DQB1*06:09 g	0.011344	0.0116
*12*	DRB1*11:01 g	0.036585	0.0332	DQB1*05:02 g	0.009926	0.0096
*13*	DRB1*04:04 g	0.036302	0.0444	DQB1*06:01 g	0.006807	0.0085
*14*	DRB1*16:02 g	0.031197	0.0427	DQB1*03:04 g	0.001418	0.0017
*15*	DRB1*11:04 g	0.019285	0.0198	DQB1*03:05 g	0.000567	0.0010
*16*	DRB1*10:01 g	0.016733	0.0178	DQB1*03:14	0.000567	0.0
*17*	DRB1*04:11	0.016449	0.0188	DQB1*03:72	0.000284	0.0
*18*	DRB1*14:01 g	0.016166	0.0185	DQB1*05:04 g	0.000284	0.0003
*19*	DRB1*01:03 g	0.013897	0.0082	DQB1*06:08	0.000284	0.0
*20*	DRB1*04:02 g	0.013897	0.0106	DQB1*06:11	0.000284	0.0

Both **Tables 1**, **2**, include the AFs obtained previously by the UCB Bank from Bogotá, as we expected, by increasing the number of samples and the cities where were collected, we found a higher number of alleles, except for HLA-DRB1. In terms of the AFs, the most common alleles remain the same in both studies, however, some frequencies and their orders change slightly.

**Figure 3:**
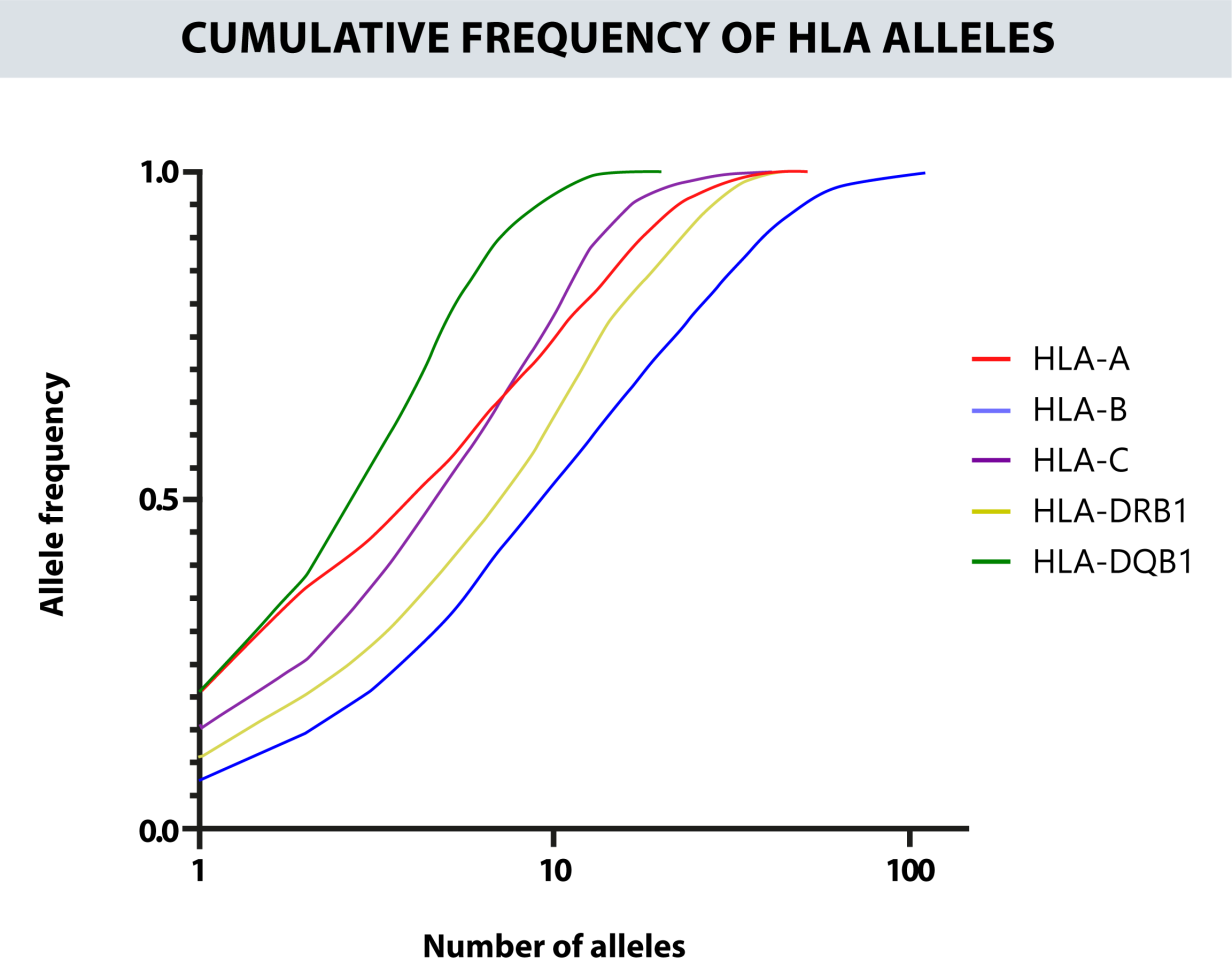
Cumulative frequency of the alleles for each HLA locus. The closer the curve is to the upper left end, the more homogeneous the locus will be. The x-axis is on a logarithmic scale. The most polymorphic locus is HLA-B, followed by HLA-DRB1 and HLA-A, and the least polymorphic is HLA-DQB1.

The cumulative frequency of each locus for the first 20 alleles was 92.91% for the HLA-A, 72.97% for the HLA-B, 97.36% for the HLA-C, 86.59% for the HLA-DRB1, and 100% for HLA-DQB1 (**Figure 3**). The zygosity is similar between the different HLA loci. Most are heterozygous, especially in HLA-B where the percent of heterozygous reach 96% of the results. On the other hand, the HLA locus that shows more homozygosity is HLA-DRB1 with a 14% (**Supplementary Figure 2**).

Finally, we compared the eleven most frequent HLA alleles between the 1347 women and 416 men to evaluate if gender might be influencing HLA segregation and how the AFs could be altered by it. The ratio of male/female is close to one in most of the cases for the 5 HLA loci, except for the alleles A*03:01g (ratio 0,703) and DQB1*05:03g (ratio 0,648) where the percent of males is meaningfully lower than females and, contrary, alleles DRB1*14:02g (ratio 1,357) and DRB1*08:02g (ratio 1,250) have a higher proportion of males than females (**Supplementary Table 3**).

### 3.2 Haplotype frequency

1626 different A~B~C~DQB1~DRB1 haplotypes were estimated with a frequency above 1e^-6^ among the 1763 typed samples. The haplotype A*24:02g~B*35:43g~C*01:02g~DQB1*03:02g~DRB1*04:07g occurred at the highest frequency (3.33%). There were 4 other haplotypes with a frequency higher than 1%:

A*29:02g~B*44:03g~C*16:01g~DQB1*02:01g~DRB1*07:01g (2.04%), A*01:01g~B*08:01g~C*07:01g~DQB1*02:01g~DRB1*03:01g (1.83%), A*33:01g~B*14:02g~C*08:02g~DQB1*05:01g~DRB1*01:02g (1.13%), and A*03:01g~B*07:02g~C*07:02g~DQB1*06:02g~DRB1*15:01g (1.05%). The 20 most frequent haplotypes are summarized in **Table 3**, and the haplotypes with a frequency higher than 0.1% are listed in **Supplementary Table 4**. In addition, in **Table 3** we compared these 20 most frequent haplotypes with the haplotype data of the UCB Bank of Bogotá and the most frequent haplotypes registered in the United States Bone Marrow Registry (USA NMDP). Haplotypic frequencies of 4 different ethnic groups registered in the USA NMDP (Caucasian, African American, Middle Eastern and North African, and Hispanic from Central and South America), were accessed *via* the Internet portal, allelefrequiencies.net. Our 20 most frequent haplotypic frequencies are in the haplotypic frequencies reported in the UCB Bank of Bogota, although the frequencies are similar in both studies, the inclusion of records from other Colombian cities generates slight changes in haplotypic frequencies due to possible regional haplotypes not represented in Bogotá. On the other hand, it is remarkable, when we compare our most frequent haplotypes in the USA NMDP records for the 4 ethnic groups chosen, only 9 of the 20 haplotypes are found in these populations, even in the Hispanic population where we would expect similar results. The most frequent haplotype A*24:02g~B*35:43g~C*01:02g~DQB1*03:02g~DRB1*04:07g is much lower in all populations, even in the Hispanic population where the difference is almost 3 percentage points. The frequencies of the following 4 frequent haplotypes have similar proximity as expected to the Hispanic population.

**Table 3:** The 20 most frequent A~B~C~DQB1~DRB1 haplotypes were identified for the donors from the Colombian BM Donor Registry.

Position	Haplotype	Frequency (n=1763)	Frequency CBB (n=1463)	Frequency arab* Middle Eastern or North Coast of Africa (n=70890)	Frequency European Caucasian (n=1242890)	Frequency African American (n=416581)	Frequency Hispanic South or Central American (n=146714)
*1*	A*24:02g~B*35:43g~C*01:02g~DQB1*03:02g~DRB1*04:07g	3,3355	4,1444	0,0012	0,0005	0,0007	0,6353
*2*	A*29:02g~B*44:03g~C*16:01g~DQB1*02:01g~DRB1*07:01g	2,0354	1,8769	0,6766	1,5671	0,3570	1,6788
*3*	A*01:01g~B*08:01g~C*07:01g~DQB1*02:01g~DRB1*03:01g	1,8286	0,9958	2,1943	6,5258	1,1238	1,5860
*4*	A*33:01g~B*14:02g~C*08:02g~DQB1*05:01g~DRB1*01:02g	1,1344	0,4785	1,1193	0,4423	0,0650	0,8749
*5*	A*03:01g~B*07:02g~C*07:02g~DQB1*06:02g~DRB1*15:01g	1,0477	1,2282	1,0365	3,1137	0,6359	1,0472
*6*	A*24:02g~B*40:02g~C*03:05g~DQB1*03:02g~DRB1*04:07g	0,9085	0,8211	n.d.	n.d.	n.d.	n.d.
*7*	A*24:02g~B*35:43g~C*01:02g~DQB1*04:02g~DRB1*08:02g	0,8574	1,0611	n.d.	n.d.	n.d.	n.d.
*8*	A*24:02g~B*35:12g~C*04:01g~DQB1*03:01g~DRB1*16:02g	0,8444	0,8697	n.d.	n.d.	n.d.	n.d.
*9*	A*03:01g~B*35:01g~C*04:01g~DQB1*05:01g~DRB1*01:01g	0,7809	0,6138	0,5446	1,1212	0,2441	0,5057
*10*	A*24:02g~B*07:02g~C*07:02g~DQB1*06:02g~DRB1*15:01g	0,7521	0,3600	0,3151	0,7311	0,1534	0,3856
*11*	A*30:02g~B*18:01g~C*05:01g~DQB1*02:01g~DRB1*03:01g	0,7087	0,9553	0,2473	0,3524	0,1026	0,6762
*12*	A*02:01g~B*07:02g~C*07:02g~DQB1*05:01g~DRB1*01:03g	0,6517	0,3418	n.d.	n.d.	n.d.	n.d.
*13*	A*24:02g~B*35:12g~C*04:01g~DQB1*03:02g~DRB1*04:07g	0,5758	0,7709	n.d.	n.d.	n.d.	n.d.
*14*	A*02:01g~B*18:01g~C*05:01g~DQB1*02:01g~DRB1*03:01g	0,5360	0,3434	n.d.	n.d.	n.d.	n.d.
*15*	A*02:01g~B*07:02g~C*07:02g~DQB1*06:02g~DRB1*15:01g	0,5247	0,5203	0,6234	1,9739	0,3540	0,5800
*16*	A*02:13~B*51:01g~C*15:02g~DQB1*03:02g~DRB1*04:04g	0,5067	0,6741	n.d.	n.d.	n.d.	n.d.
*17*	A*02:01g~B*44:02g~C*05:01g~DQB1*06:03g~DRB1*13:01g	0,4991	0,2614	n.d.	n.d.	n.d.	n.d.
*18*	A*24:02g~B*35:43g~C*01:02g~DQB1*03:01g~DRB1*14:02g	0,4812	0,2083	n.d.	n.d.	n.d.	n.d.
*19*	A*02:01g~B*38:01g~C*12:03g~DQB1*06:03g~DRB1*13:01g	0,4458	0,3003	n.d.	n.d.	n.d.	n.d.
*20*	A*02:01g~B*44:02g~C*05:01g~DQB1*05:01g~DRB1*01:01g	0,4446	0,6375	n.d.	n.d.	n.d.	n.d.

* n.d. No data is available in the portal allelefrequencies.net.

Our haplotype frequencies were compared with de UCB Bank of Bogotá and 4 different ethnic groups frequencies from USA NMDP registered on the website allelefrequencies.net. The data is shown in percent (%).

**Figure 4:**
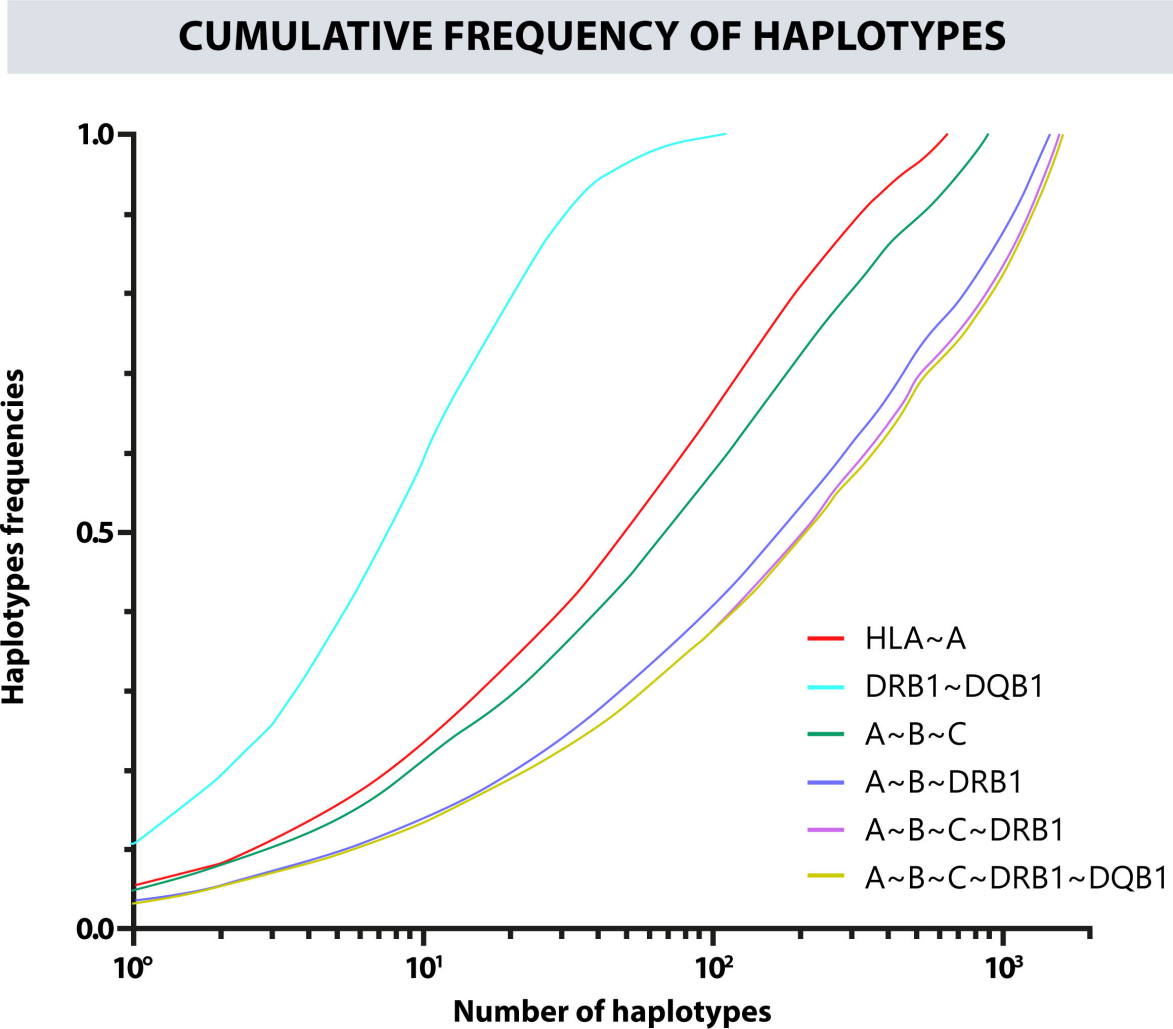
Cumulative frequency of the haplotypes for the different combinations of classic HLA loci.

**Figure 5:**
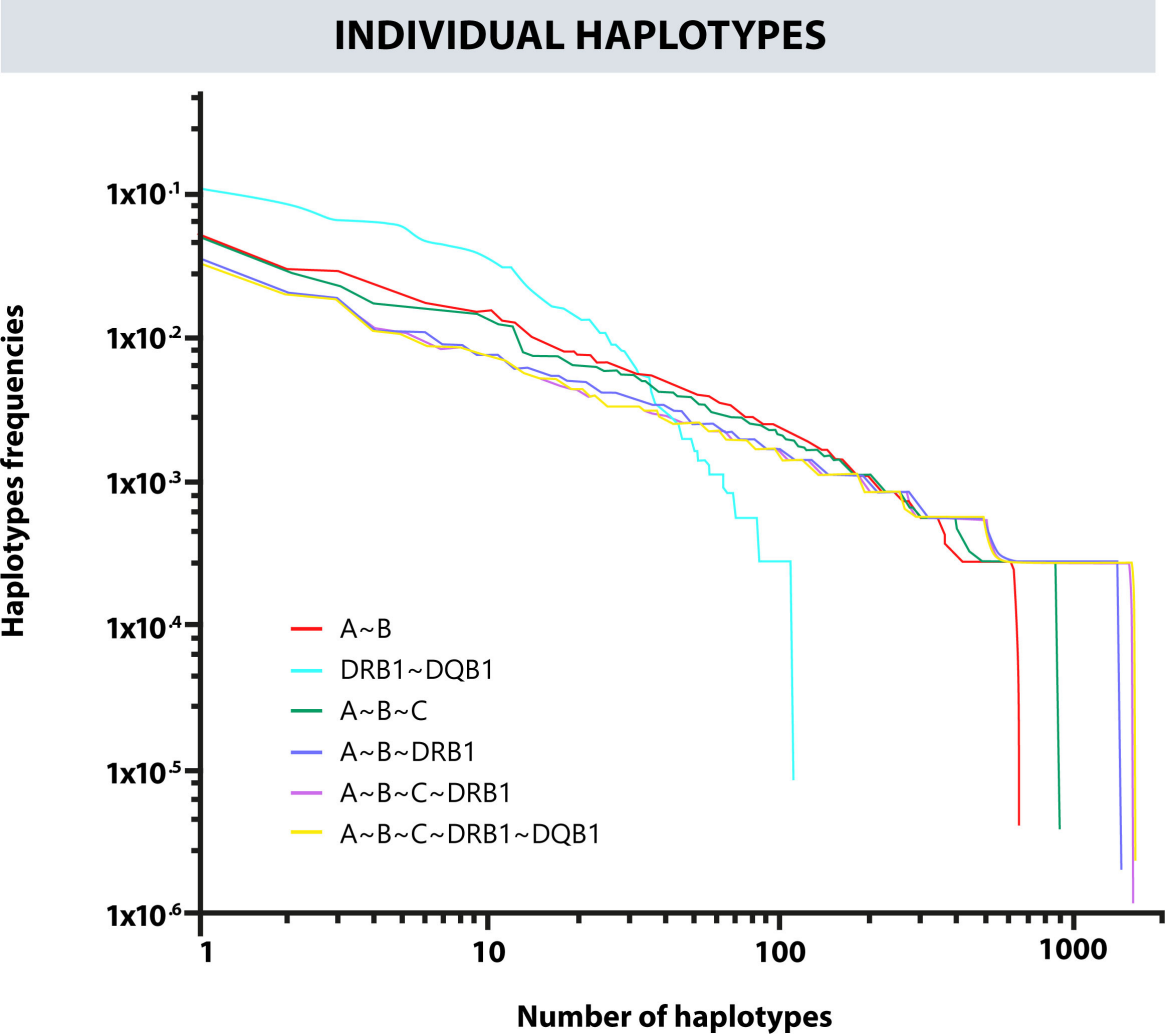
Frequency variation in each observed haplotype and their variation with different combinations of loci.

Likewise, a frequency estimation of haplotypes A~B, A~B~C, A~B~DRB1, DRB1~DQB1, and A~B~C~DRB1 was performed. The Cumulative frequencies for each haplotype are shown in **Figure 4**. The most frequent haplotypes were A*24:02g~B*35:43g (5.23%), A*29:02g~B*44:03g (3.03%), A*24:02g~B*40:02g (2.89%); A*24:02g~B*35:43g~C*01:02g (5.09%), A*29:02g~B*44:03g~C*16:01g (2.94%), A*01:01g~B*08:01g~C*07:01g (2.31%); A*24:02g~B*35:43g~DRB1*04:07g (3.45%), A*29:02g~B*44:03g~DRB1*07:01g (2.07%), A*01:01g~B*08:01g~DRB1*03:01g (1.85%); A*24:02g~B*35:43g~C*01:02g~DRB1*04:07g (3.34%), A*29:02g~B*44:03g~C*16:01g~DRB1*07:01g (2.03%), A*01:01g~B*08:01g~C*07:01g~DRB1*03:01g (1.82%); DQB1*03:02g~DRB1*04:07g (10.86%), DQB1*02:01g~DRB1*07:01g (8.43%), and DQB1*04:02g~DRB1*08:02g (6.63%). **Figure 5** shows the observed frequencies for each of the estimated haplotypes and the variation with the different combinations of loci.

**Table 4** provides the observed and expected heterozygosity, p-value, and standard deviation. Considering the allelic distributions of the HLA-B, HLA-C, HLA-DRB1, and HLA-DQB1 loci of the donors, the genotypic frequencies met HWE (p > 0.05); however, the distribution of the HLA-A locus showed significant deviation (p = 0.01440). In addition, LD was detected between HLA class I loci as well as class II loci (**Supplementary Table 5**).

**Table 4:** Hardy-Weinberg equilibrium (HWE) for the 1763 donors.

Locus	Genotypes	Observed heterozygosity	Expected heterozygosity	p-value	Standard deviation (SD)
*HLA-A*	1763	0.91435	0.90716	0.01440*	0.00005
*HLA-B*	1763	0.95973	0.96407	0.71737	0.00009
*HLA-C*	1763	0.91548	0.92209	0.08922	0.00012
*HLA-DRB1*	1763	0.94385	0.94786	0.55199	0.00016
*HLA-DQB1*	1763	0.86330	0.86292	0.31754	0.00032

Statistical significance (p < 0.05).

### 3.3 Detection of new alleles

Ten new HLA alleles were identified and submitted to the IPD-IMGT/HLA database. These alleles were named by the nomenclature committee of the World Health Organization (WHO). Seven new HLA alleles were described for class I; 6 of the 7 alleles, had a substitution of a nitrogenous base, generating an amino acid change, the remaining new allele being a synonymous substitution. Most of the substitutions occurred in exon 3 (4 changes), exon 4 (2 changes), and exon 2 (1 change). For HLA class II alleles, 3 changes occurred in exon 3, 2 of these substitutions generated an amino acid change; the remaining is a null allele generated by a 2-base deletion that causes a change in the reading frame, producing a premature stop codon (**Table 5**).

**Table 5:** New HLA alleles submitted currently.

New allele	Reference allele	SNP	Exon	Change from aa	GenBank AN
A*24:487	A*24:02:01	G-> T	3	Pro -> Ala	MG967421
B*30:172	A*30:01:01:01	C-> G	3	Ala -> Pro	MT364235
B*35:475	B*35:43:01	A -> T	3	Arg-> Trp	MN623624
B*35:476	B*35:43:01	G-> A	2	Ala -> Thr	MN623625
B*14:91	B*14:02:01:01	G-> A	4	Val -> Met	MN840506
B*39:165	B*39:05:01:01	G-> A	4	Glu -> Lys	MN840507
B*35:43:04	B*35:43:01	G-> A	3	Gly -> Gly	MN508229
DRB1*14:02:09	DRB1*14:02:01:02	G-> A	3	Glu -> Glu	MN840498
DRB1*04:315	DRB1*04:07:01:02	C -> G	3	Leu -> Val	MN508233
DRB1*13:298N	DRB1*13:02:01:01	del_CA	3	Change in reading frame with a premature stop codon	MN508231

### 3.4 AFs of class II loci and extended haplotypes

488 donor samples were typed at high resolution by DKMS Life Science Lab (Dresden, Germany) for classic HLA loci, including class II loci (HLA-DPB1, HLA-DPA1, and HLA-DQA1). The frequencies observed for each of these class II loci are summarized in **Table 6**. Among the donor samples, DRB3*02:02:01G (15.57%), DRB3*01:01:02G (14.14%), DRB3* 03:01:01G (6.04%), DRB4*01:01:01G (32.07%), and DRB5*01:01:01G (6.97%) occurred at a frequency higher than 5% (**Supplementary Table 6**). Subsequently, HFs were estimated, including the HLA-DPB1, HLA-DPA1, and HLA-DQA1 loci, generating an extended haplotype of 8 HLA loci for the donors. A total of 733 extended haplotypes were estimated, and the 20 most frequent haplotypes are summarized in **Table 7**, with the most frequent haplotype being A*24:02g~B*35:43g~C*01:02g~DPA1*02:01g~DPB1*14:01g~DQA1*03:01g~DQB1*03:02g~DRB1*04:07g, with a frequency of 1.437%.

**Table 6:** Allele frequencies for class II, HLA-DPB1, HLA-DPA1, and HLA-DQA1 loci.

HLA-DPB1	Frequency	HLA-DPA1	Frequency	HLA-DQA1	Frequency
DPB1*04:01 g	0.26899	DPA1*01:03 g	0.69713	DQA1*05:01 g	0.24230
DPB1*04:02 g	0.22382	DPA1*02:01 g	0.23922	DQA1*03:01 g	0.23306
DPB1*02:01 g	0.11807	DPA1*02:02 g	0.04826	DQA1*01:02 g	0.14476
DPB1*14:01 g	0.08522	DPA1*03:01 g	0.01129	DQA1*01:01 g	0.13758
DPB1*01:01 g	0.06468	DPA1*01:04	0.00205	DQA1*02:01 g	0.10472
DPB1*03:01 g	0.06058	DPA1*04:01 g	0.00103	DQA1*04:01 g	0.06571
DPB1*13:01 g	0.03491	DPA1*01:05 g	0.00103	DQA1*01:03 g	0.06366
DPB1*11:01 g	0.03080			DQA1*01:10	0.00308
DPB1*17:01 g	0.03080			DQA1*06:01 g	0.00308
DPB1*05:01 g	0.01643			DQA1*05:02	0.00205
DPB1*18:01 g	0.01129				
DPB1*02:02 g	0.01027				
DPB1*06:01 g	0.00821				
DPB1*10:01 g	0.00821				
DPB1*23:01 g	0.00513				
DPB1*15:01 g	0.00411				
DPB1*19:01 g	0.00411				
DPB1*09:01 g	0.00308				
DPB1*16:01 g	0.00308				
DPB1*34:01 g	0.00205				
DPB1*153:01	0.00103				
DPB1*24:01	0.00103				
DPB1*30:01	0.00103				
DPB1*41:01	0.00103				
DPB1*79:01	0.00103				
DPB1*85:01 g	0.00103				

**Table 7:** 20 extended haplotypes of the 8 most frequent classic HLA loci.

Position	Haplotype	Frequency
*1*	A*24:02g~B*35:43g~C*01:02g~DPA1*02:01g~DPB1*14:01g~DQA1*03:01g~DQB1*03:02g~DRB1*04:07g	0.01437
*2*	A*03:01g~B*35:01g~C*04:01g~DPA1*01:03g~DPB1*04:02g~DQA1*01:01g~DQB1*05:01g~DRB1*01:01g	0.00924
*3*	A*24:02g~B*07:02g~C*07:02g~DPA1*01:03g~DPB1*04:01g~DQA1*01:02g~DQB1*06:02g~DRB1*15:01g	0.00909
*4*	A*29:02g~B*44:03g~C*16:01g~DPA1*01:03g~DPB1*04:01g~DQA1*02:01g~DQB1*02:01g~DRB1*07:01g	0.00616
*5*	A*24:02g~B*40:02g~C*03:04g~DPA1*01:03g~DPB1*04:02g~DQA1*05:01g~DQB1*03:01g~DRB1*03:02g	0.00513
*6*	A*02:01g~B*44:02g~C*05:01g~DPA1*02:01g~DPB1*17:01g~DQA1*01:01g~DQB1*05:01g~DRB1*01:01g	0.00513
*7*	A*11:01g~B*53:01g~C*04:01g~DPA1*01:03g~DPB1*04:01g~DQA1*01:02g~DQB1*06:04g~DRB1*13:02g	0.00513
*8*	A*24:02g~B*35:02g~C*04:01g~DPA1*01:03g~DPB1*02:01g~DQA1*05:01g~DQB1*02:01g~DRB1*03:01g	0.00513
*9*	A*02:01g~B*18:01g~C*05:01g~DPA1*01:03g~DPB1*02:01g~DQA1*05:01g~DQB1*02:01g~DRB1*03:01g	0.00513
*10*	A*33:01g~B*14:02g~C*08:02g~DPA1*01:03g~DPB1*04:01g~DQA1*01:01g~DQB1*05:01g~DRB1*01:02g	0.00513
*11*	A*24:02g~B*40:02g~C*03:05g~DPA1*01:03g~DPB1*04:02g~DQA1*03:01g~DQB1*03:02g~DRB1*04:07g	0.00513
*12*	A*24:02g~B*35:43g~C*01:02g~DPA1*01:03g~DPB1*04:01g~DQA1*04:01g~DQB1*04:02g~DRB1*08:02g	0.00508
*13*	A*24:02g~B*40:02g~C*03:05g~DPA1*02:01g~DPB1*14:01g~DQA1*03:01g~DQB1*03:02g~DRB1*04:07g	0.00411
*14*	A*02:01g~B*39:11~C*07:02g~DPA1*01:03g~DPB1*04:02g~DQA1*04:01g~DQB1*04:02g~DRB1*08:02g	0.00411
*15*	A*02:13~B*51:01g~C*15:02g~DPA1*01:03g~DPB1*04:02g~DQA1*05:01g~DQB1*03:01g~DRB1*16:02g	0.00411
*16*	A*03:01g~B*07:02g~C*07:02g~DPA1*01:03g~DPB1*02:01g~DQA1*01:02g~DQB1*06:02g~DRB1*15:01g	0.00411
*17*	A*29:02g~B*57:01g~C*07:01g~DPA1*01:03g~DPB1*04:01g~DQA1*01:01g~DQB1*05:01g~DRB1*01:01g	0.00411
*18*	A*26:01g~B*38:01g~C*12:03g~DPA1*01:03g~DPB1*04:01g~DQA1*01:01g~DQB1*05:03g~DRB1*14:01g	0.00411
*19*	A*30:02g~B*18:01g~C*05:01g~DPA1*01:03g~DPB1*02:02g~DQA1*05:01g~DQB1*02:01g~DRB1*03:01g	0.00411
*20*	A*03:01g~B*07:02g~C*07:02g~DPA1*01:03g~DPB1*04:01g~DQA1*01:02g~DQB1*06:02g~DRB1*15:01g	0.00411

### 3.5 Distribution of HLA alleles and HF in Colombia

**Figure 6:**
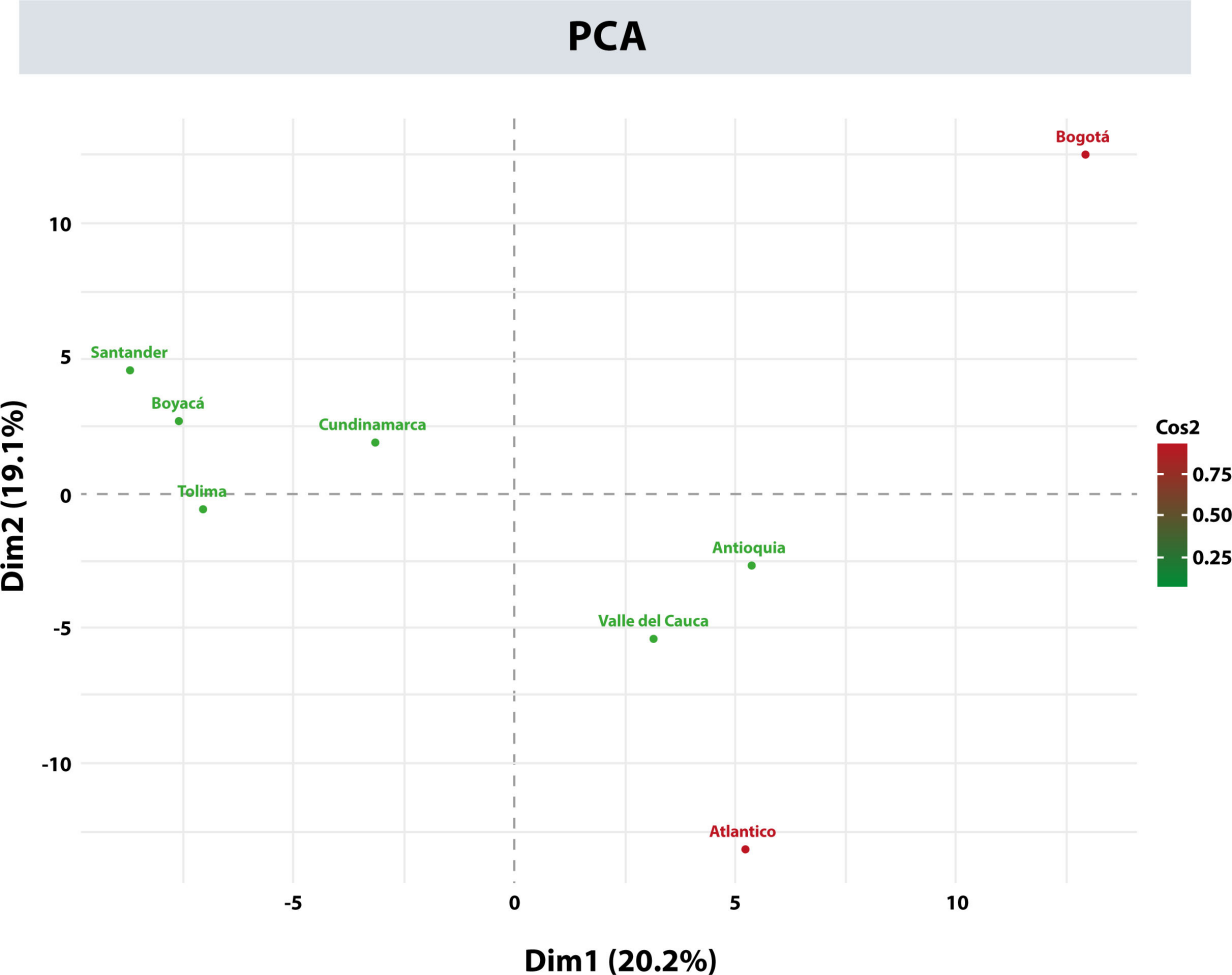
Principal component analysis (PCA) with allele frequencies for the departments of Antioquia, Valle del Cauca, Atlántico, Cundinamarca, Boyacá, Santander, Tolima, and Bogotá.

Within the project aiming to create a national BM Donor Registry in Colombia, a donor recruitment program was established in the 4 main cities of the country: Bogotá, Medellín, Cali, and Barranquilla. This program’s purpose was to increase the representation of the HLA alleles in the Registry and gain an overall view of the allelic distribution throughout the different regions in Colombia. For this analysis, the AFs of the HLA-A, -B, -C, -DRB1, and -DQB1 loci of the donors from the departments with the highest number of records were included, i.e., Antioquia, Valle del Cauca, Atlántico, Cundinamarca, Boyacá, Santander, Tolima, and Bogotá. **Table 8** shows the 11 most frequent alleles for each HLA locus in Bogotá, which is the city with most of the records. These frequencies were compared by departments regarding their frequency position, for each population. Using the HLA AFs of the 5 loci for each department, principal component analysis (PCA) was performed to determine whether the distribution of these frequencies would allow differentiating the populations (**Figure 6**). Populations were categorized into 3 groups using PCA: Bogotá was alone in the first group; donors from Tolima, Boyacá, Santander, and Cundinamarca were in the second group; and the population from Atlántico, Antioquia, and Valle del Cauca, which were more dispersed, were in the third group.

**Table 8:** The 11 most frequent HLA-A, HLA-B, HLA-C, HLA-DRB1, and HLA-DQB1 alleles in the city of Bogotá and the rank that these alleles occupied in the departments of Antioquia, Valle del Cauca, Atlántico, Cundinamarca, Boyacá, Santander and Tolima.

HLA-A	Bogotá		Antioquia		Valle del Cauca		Atlántico		Cundinamarca		Boyacá		Santander		Tolima		
Allele	Rank	Frequency	Rank	Frequency	Rank	Frequency	Rank	Frequency	Rank	Frequency	Rank	Frequency	Rank	Frequency	Rank		Frequency
A*24:02 g	1	0.2181	1	0.1810	1	0.1926	1	0.1798	1	0.2286	1	0.2561	1	0.2857	1		0.2286
A*02:01 g	2	0.1624	2	0.1687	2	0.1639	2	0.1404	2	0.1571	2	0.1341	2	0.1571	2		0.1286
A*01:01 g	3	0.0725	5	0.0521	3	0.0943	5	0.0789	4	0.0714	3	0.0854	3	0.0857	3		0.1000
A*03:01 g	4	0.0633	3	0.0859	4	0.0615	3	0.0965	5	0.0571	4	0.0610	4	0.0857	4		0.0857
A*29:02 g	5	0.0536	8	0.0368	5	0.0492	6	0.0482	3	0.0786	7	0.0366	6	0.0571	5		0.0714
A*11:01 g	6	0.0434	4	0.0583	7	0.0369	8	0.0395	7	0.0429	5	0.0610	7	0.0429	7		0.0429
A*68:01 g	7	0.0429	6	0.0429	9	0.0287	4	0.0965	6	0.0500	8	0.0366	10	0.0286	6		0.0571
A*26:01 g	8	0.0337	7	0.0399	8	0.0328	7	0.0439	13	0.0143	13	0.0244	18	0.0000	14		0.0286
A*02:22 g	9	0.0312	12	0.0215	13	0.0246	15	0.0132	8	0.0357	17	0.0122	5	0.0714	9		0.0286
A*31:01 g	10	0.0296	9	0.0368	6	0.0410	9	0.0351	10	0.0214	12	0.0244	8	0.0429	8		0.0429
A*23:01 g	11	0.0286	10	0.0368	10	0.0287	10	0.0351	11	0.0214	9	0.0366	11	0.0286	11		0.0286

*HLA-B*	*Bogotá*		*Antioquia*		*Valle del Cauca*		*Atlántico*		*Cundinamarca*		*Boyacá*		*Santander*		*Tolima*		
*Allele*	*Rank*	*Frequency*	*Rank*	*Frequency*	*Rank*	*Frequency*	*Rank*	*Frequency*	*Rank*	*Frequency*	*Rank*	*Frequency*	*Rank*	*Frequency*	*Rank*		*Frequency*
B*35:43 g	1	0.0909	2	0.0675	2	0.0574	12	0.0263	2	0.1000	1	0.1707	1	0.1143	6		0.0429
B*40:02 g	2	0.0756	5	0.0521	1	0.0820	2	0.0658	1	0.1071	6	0.0488	5	0.0571	2		0.0714
B*51:01 g	3	0.0633	6	0.0521	5	0.0451	8	0.0351	11	0.0286	5	0.0610	9	0.0429	1		0.0857
B*44:03 g	4	0.0618	7	0.0521	4	0.0574	4	0.0439	3	0.0786	2	0.0976	2	0.0714	3		0.0571
B*07:02 g	5	0.0557	8	0.0491	9	0.0369	7	0.0395	4	0.0571	3	0.0610	4	0.0571	5		0.0571
B*14:02 g	6	0.0506	1	0.0828	7	0.0410	9	0.0351	7	0.0500	7	0.0488	13	0.0286	8		0.0429
B*35:01 g	7	0.0501	3	0.0675	8	0.0410	1	0.0789	5	0.0500	10	0.0366	3	0.0714	4		0.0571
B*08:01 g	8	0.0358	17	0.0184	6	0.0451	3	0.0570	10	0.0286	8	0.0488	14	0.0286	12		0.0286
B*44:02 g	9	0.0332	9	0.0184	11	0.0287	10	0.0351	9	0.0357	NA	NA	15	0.0286	13		0.0286
B*39:05 g	10	0.0301	24	0.0123	24	0.0123	14	0.0263	22	0.0143	21	0.0122	8	0.0571	11		0.0286
B*18:01 g	11	0.0281	4	0.0614	3	0.0574	6	0.0263	8	0.0429	4	0.0610	12	0.0286	7		0.0429

*HLA-C*	*Bogotá*		*Antioquia*		*Valle del Cauca*		*Atlántico*		*Cundinamarca*		*Boyacá*		*Santander*		*Tolima*		
*Allele*	*Rank*	*Frequency*	*Rank*	*Frequency*	*Rank*	*Frequency*	*Rank*	*Frequency*	*Rank*	*Frequency*	*Rank*	*Frequency*	*Rank*	*Frequency*	*Rank*		*Frequency*
C*04:01 g	1	0.1512	1	0.2025	1	0.1270	1	0.1272	1	0.1571	2	0.1585	1	0.1571	1		0.1286
C*01:02 g	2	0.1195	4	0.0798	3	0.1107	7	0.0570	2	0.1214	1	0.2195	2	0.1429	8		0.0571
C*07:02 g	3	0.1083	2	0.1043	4	0.0984	3	0.0921	3	0.1071	4	0.1098	4	0.1143	2		0.1000
C*07:01 g	4	0.0863	5	0.0798	2	0.1189	2	0.1184	5	0.0714	3	0.1220	5	0.0857	3		0.0857
C*03:04 g	5	0.0827	7	0.0552	5	0.0984	5	0.0614	4	0.0857	6	0.0488	3	0.1286	4		0.0714
C*08:02 g	6	0.0582	3	0.0951	8	0.0492	8	0.0570	6	0.0714	7	0.0488	12	0.0143	6		0.0714
C*16:01 g	7	0.0562	8	0.0429	11	0.0410	12	0.0439	9	0.0571	8	0.0488	6	0.0857	5		0.0714
C*06:02 g	8	0.0485	9	0.0368	6	0.0615	6	0.0614	11	0.0286	11	0.0366	11	0.0286	10		0.0571
C*05:01 g	9	0.0429	6	0.0798	7	0.0533	9	0.0526	8	0.0571	12	0.0244	8	0.0571	9		0.0571
C*15:02 g	10	0.0419	14	0.0215	14	0.0205	11	0.0482	14	0.0143	5	0.0610	10	0.0286	12		0.0429
C*02:02 g	12	0.0317	10	0.0368	12	0.0287	4	0.0746	10	0.0429	10	0.0366	NA	NA	7		0.0714

*HLA-DQB1*	*Bogotá*		*Antioquia*		*Valle del Cauca*		*Atlántico*		*Cundinamarca*		*Boyacá*		*Santander*		*Tolima*		
*Allele*	*Rank*	*Frequency*	*Rank*	*Frequency*	*Rank*	*Frequency*	*Rank*	*Frequency*	*Rank*	*Frequency*	*Rank*	*Frequency*	*Rank*	*Frequency*	*Rank*		*Frequency*
DQB1*03:02 g	1	0.2227	2	0.1748	1	0.1721	2	0.1667	1	0.2571	1	0.2195	1	0.2429	1		0.2143
DQB1*03:01 g	2	0.1716	3	0.1564	2	0.1721	1	0.2544	2	0.1571	4	0.0976	2	0.1857	3		0.1286
DQB1*02:01 g	3	0.1542	1	0.2147	3	0.1598	3	0.1535	3	0.1357	2	0.2195	3	0.1714	2		0.2143
DQB1*04:02 g	4	0.1267	5	0.1074	5	0.0656	6	0.0658	4	0.1357	5	0.0976	4	0.1429	6		0.0714
DQB1*05:01 g	5	0.1139	4	0.1288	4	0.1557	4	0.1228	5	0.1143	3	0.1707	6	0.0571	4		0.1286
DQB1*06:02 g	6	0.0618	6	0.0675	6	0.0615	5	0.0921	6	0.0857	6	0.0976	8	0.0286	5		0.0857
DQB1*06:03 g	7	0.0475	7	0.0521	7	0.0615	7	0.0439	7	0.0571	7	0.0610	7	0.0571	7		0.0571
DQB1*03:03 g	8	0.0276	9	0.0184	8	0.0410	10	0.0132	9	0.0143	9	0.0122	NA	NA	8		0.0286
DQB1*06:04 g	9	0.0240	8	0.0399	9	0.0287	8	0.0439	8	0.0143	8	0.0122	5	0.0714	10		0.0143
DQB1*05:03 g	10	0.0194	10	0.0123	10	0.0246	9	0.0219	10	0.0071	NA	NA	NA	NA	NA		NA
DQB1*06:09 g	11	0.0117	11	0.0123	13	0.0123	11	0.0088	11	0.0071	NA	NA	10	0.0143	11		0.0143

*HLA-DRB1*	*Bogotá*		*Antioquia*		*Valle del Cauca*		*Atlántico*		*Cundinamarca*		*Boyacá*		*Santander*		*Tolima*		
*Allele*	*Rank*	*Frequency*	*Rank*	*Frequency*	*Rank*	*Frequency*	*Rank*	*Frequency*	*Rank*	*Frequency*	*Rank*	*Frequency*	*Rank*	*Frequency*	*Rank*		*Frequency*
DRB1*04:07 g	1	0.1267	2	0.0920	2	0.0820	3	0.0570	1	0.1286	1	0.1220	1	0.1286	3		0.0857
DRB1*07:01 g	2	0.0981	1	0.1258	1	0.0984	2	0.0702	4	0.0786	2	0.1098	3	0.1000	1		0.1286
DRB1*08:02 g	3	0.0797	7	0.0460	10	0.0410	21	0.0132	3	0.0857	5	0.0854	2	0.1143	5		0.0714
DRB1*03:01 g	4	0.0623	3	0.0890	5	0.0615	1	0.0789	9	0.0429	4	0.1098	5	0.0714	2		0.1000
DRB1*15:01 g	5	0.0618	8	0.0460	6	0.0615	5	0.0570	5	0.0786	3	0.1098	11	0.0286	4		0.0857
DRB1*04:05 g	6	0.0501	9	0.0429	16	0.0205	14	0.0307	2	0.0929	13	0.0122	17	0.0143	NA		NA
DRB1*13:01 g	7	0.0470	5	0.0521	4	0.0697	10	0.0482	8	0.0500	8	0.0488	7	0.0571	6		0.0571
DRB1*14:02 g	8	0.0455	11	0.0399	8	0.0533	13	0.0351	14	0.0214	14	0.0122	6	0.0714	9		0.0429
DRB1*01:01 g	9	0.0383	4	0.0552	3	0.0820	4	0.0570	10	0.0429	9	0.0488	16	0.0143	8		0.0571
DRB1*13:02 g	10	0.0378	6	0.0521	9	0.0451	11	0.0482	16	0.0143	15	0.0122	4	0.0857	12		0.0286
DRB1*01:02 g	11	0.0373	10	0.0429	7	0.0533	12	0.0351	12	0.0357	7	0.0854	9	0.0429	10		0.0429

The A~B~C~DQB1~DRB1 haplotypes from each department were also estimated. The most frequent haplotypes are compiled in **Supplementary Table 7**. Notably, the A*24:02g~B*35:43g~C*01:02g~DQB1*03:02g~DRB1*04:07g haplotype was the most frequent in all departments except for Atlántico, Santander, and Tolima.

## 4 Discussion

This study is part of the initiative led in the last 5 years by the IDCBIS, which aims to create and consolidate a National BM Donor Registry in Colombia, willing to provide citizens with an affordable therapeutic option for allogeneic HSC transplant because this alternative cannot always be considered due to economic, medical or logistical reasons. Despite the limitations associated with the sample size, the results and analyses in this work are intended to have a better understanding of the genetic diversity in Colombia. This is the largest high-resolution study for classic HLA loci, including donors recruited in the 4 main cities of the country: Bogotá, Medellín, Cali, and Barranquilla carried out by the Colombian BM Donor Registry.

Páez-Gutiérrez et al. (47) reported a number of similar alleles in Bogotá to those in our sample. A slight increase in the number of alleles was observed for the HLA-A (+1), HLA-C (+5), and HLA-DQB1 (+2) loci, and a striking increase was observed for the most polymorphic locus HLA-B (+13). The class II locus, HLA-DRB1 (-4) was the only one with a reduction in the number of alleles observed. In both studies, alleles A*24:02g (20.87 - 0.06%), B*35:43g (7.69 + 0.96%), C*04:01g (15.40 – 0,50%), DRB1*04:07g (11.03 + 1.24%) and DQB1*03:02g (20.96 + 1.49%) were the most frequent. For the A~B~C~DQB1~DRB1 haplotypes, 187 more were detected in our study than in the study by Páez-Gutiérrez et al. (47); nevertheless, the A*24:02g~B*35:43g~C*01:02g~DQB1*03:02g~DRB1*04:07g haplotype was the most frequent in both studies, although the standard deviation was close to a percentage point (3.33 + 0.81%). The frequencies of the HLA-DRB1 and HLA-DQB1 loci and the DRB1*04:07g~DQB1*0302g haplotype in the study by Del Río-Ospina et al. (48) are consistent with our results. We also found similarities in the reported HLA alleles and their frequencies in other published studies involving the Colombian population; however, these studies were not performed in high-resolution typing (49–52).

The fact that the most frequent haplotype in our population is not represented in the compared populations from the USA NMDP, including the Hispanic population, and in addition, the absence of 11 of the 20 most frequent haplotypes compared, leads us to hypothesize that the migratory processes occurred in Colombia and the component of native groups have generated haplotypic combinations unusual or infrequent in other populations. As the size of the Colombian registry increases and its scope is extended to different regions and different populations, it will be possible to confirm whether these AFs and HFs are really particular to Colombians.

NGS used for typing the HLA loci led to the identification of 10 new HLA alleles among the 1763 samples analyzed. Most new alleles occurred in the HLA-B locus, and a null HLA-DRB1 allele was identified. The nucleotide sequences of the 10 new alleles were submitted to GenBank with the accession numbers MG967421, MT364235, MN23624, MN623625, MN840506, MN 840507, MN84508229, MN840498, MN508233, and MN508231 and were assigned the names A*24:487, B*30:172, B*:35:475, B*35:376, B*14:91, B*39:165, B*35:53:04, DRB1*14:02:09, DRB1*04:315, and DRB1*13:298N by the HLA system nomenclature committee of the WHO (53–58). The detection of these 10 alleles in a sample that represents approximately 0.01% of the Colombian population may indicate that the number of possible new alleles not reported circulating in the country is in the thousands if the trend found herein were maintained. In addition, the majority of the donors included in the registry corresponds to the mestizo population (97.41%), while the representation in the registry of other minority ethnic groups, such as Afro-American and Native American, is only 0.89% and 0.39%, respectively. Therefore, it would be expected that the identification of new HLA alleles in these minority groups will be higher as their participation in the Colombian registry increases.

The role of the HLA-DPB1 has gained greater relevance due to its association with various pathologies, including cancer (59), autoimmune diseases, such as rheumatoid arthritis (60), responses to viral infection (61), and vaccines (62, 63). Regarding stem cell transplants, there is still controversy about the inclusion of the HLA-DPB1 locus in the search algorithms for 12/12 histocompatibility (64) due to the increasing number of reports indicating that a mismatch in this locus increases the risk of developing GVHD (65). Data for HLA-DPB1 and other HLA class II loci is reported for the first time in high-resolution in Colombia. Notably, the most frequent alleles were DPB1*04:01g (26, 90%), DPB1*04:02g (22.38%), and DPB1*02:01g (11.81%); however, when analyzing the extended haplotype of the 8 most frequent loci, i.e., A*24:02g~B*35:43g~C*01:02g~DPA1*02:01g~DPB1*14:01g~DQA1*03:01g~DQB1*03:02g~DRB1*04:07g, the accompanying HLA-DPB1 allele was DPB1*14:01g, which had an AF of 8.52%. Likewise, from the analysis of the data of 488 donors, the first report of frequencies of the DRB3/4/5 alleles in Colombia was generated, with the most frequent alleles for each locus being DRB3*02:02:01G (15.57%), DRB4*01:01:01G (32.07%) and DRB5*01:01:01G (6.97%). Although these genes are not currently included in the selection of unrelated donors, there is increasing evidence of their importance in the incidence of acute GVHD (66).

Recruiting donors in several cities of Colombia allowed the registration of the HLA typing of donors from almost all departments in the national territory, except the Amazonian departments. In addition, it allowed an exploratory analysis of the behavior of AFs and HFs among the 8 departments with the most records: Antioquia, Valle del Cauca, Atlántico, Cundinamarca, Boyacá, Santander, Tolima, and Bogotá. Based on these AFs and HFs, the allele and haplotype distribution are quite heterogeneous in Colombia, especially in the department of Atlántico. In this department, the most frequent alleles and haplotypes vary significantly from those in the rest of the departments analyzed, especially the frequencies of the HLA-B, -DRB1, and -DQB1 loci.

Contrasting our data represents a challenge due to the lack of high-resolution studies of HLA loci frequencies in Colombia; nevertheless, the results published by the public UCB Bank of Bogotá (47) were similar to ours in number of alleles, and their frequencies for the city. We propose that the heterogeneous behavior observed among departments concerning AFs and HFs are mainly generated by differences in the population for each region. Increase the number of donors in the other departments might confirm this heterogeneous behavior and will be interesting to combine with other studies such as STRs or AIMs to confirm the differences in the region populations (67).

In Latin America AFs and HFs reported by countries bordering Colombia, such as Panamá (68), Venezuela (69), and Ecuador (70), showed frequencies similar to those described in our study, where alleles HLA-A*2, -A*24, -B*35, -DRB1*04 and -DQB1*0302 are the most frequent, and haplotype A*24~B*35~DRB1*04~DQB1*03:02 is the most frequent in Ecuador, Panamá, and Colombia. This coincidence may be explained by common migratory and historical processes over the centuries. In contrast, in the south of the continent the haplotype A*29~B*44~DRB1*07, the most frequent in Spain (71) and also found in other European populations, was reported. Other European haplotypes such as A*01~B*08~DRB1*03, and A*03~B*07~DRB1*15 occur frequently in Brazilian (REDOME) (72), Argentina (INCUCAI) BM Donor Registries (73), and the UCB banks from Buenos Aires (Garrahan Pediatric Hospital) (74), and Santiago de Chile (75). The presence of a greater European component in the south of the continent shows the difference in the processes of miscegenation that occurred in the north and the south.

This study is the first approach of the Colombian BM Donor Registry toward understanding the genetic diversity in Colombia, a result of migratory processes and centuries of miscegenation among different ethnic groups that have coexisted in the national territory. Although the number of registered and typed donors is still insufficient to determine this genetic variety, it is a starting point for better understanding the distribution of the alleles and haplotypes that compose the genes of the MHC in the country. This work will allow the recent Colombian BM Donor Registry to design donor recruitment strategies, both to increase the number of donors registered, and to target regions where non-common HLA alleles and haplotypes are found more frequently, to offer a therapeutic transplant option to all Colombians regardless their origin or ethnic group.

Likewise, we hope that these results will be a source of reference for subsequent studies of populations in Colombia and become a useful tool to determine the size of the national registry, moreover, allowing Colombia’s national BM Donor Registry to establish future collaborations with other centers, institutes and organizations worldwide.

## Data availability statement

The original contributions presented in the study are included in the article/**Supplementary Material**. Further inquiries can be directed to the corresponding author.

## Ethics statement

The studies involving human participants were reviewed and approved by Comité de Ética de la Secretaría Distrital de Salud. The patients/participants provided their written informed consent to participate in this study.

## Author contributions

DH-M, IP-G, and VD contributed to the initial conception of analyzing the HLA frequencies and haplotypes in Colombia and reviewing the available bibliography. NC, MM, and PC contributed with the BM Donor Registry perspective for distribution within the country. DH-M, IP-G, VD, NC, MM, and PC drafted, reviewed, and approved the submitted version of the manuscript. BC had financial support and supervised the project.
