# Supplementary material for: Distributions of the HLA-A, HLA-B, HLA-C, HLA-DRB1, and HLA-DQB1 alleles and haplotype frequencies of 1763 stem cell donors in the Colombian Bone Marrow Registry typed by next-generation sequencing
Source: Front Immunol. 2023 Jan 9;13:1057657. doi: 10.3389/fimmu.2022.1057657 (PMC9869256; doi:10.3389/fimmu.2022.1057657)
Supplement: Supplementary file 1 [file DataSheet_1.docx]

Supplementary Material

## Supplementary Figures
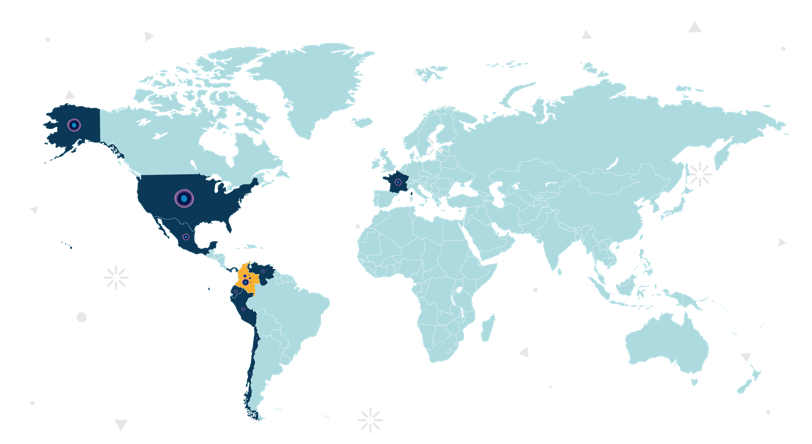
Supplementary Figure 1. The national Bone Marrow Donor Registry recruited 30 foreigners residing in Colombia, most of them from Venezuela (20) and the rest from the United States (4), Mexico (1), Panama (1), Chile (1), Peru (1), Ecuador (1) and France (1).


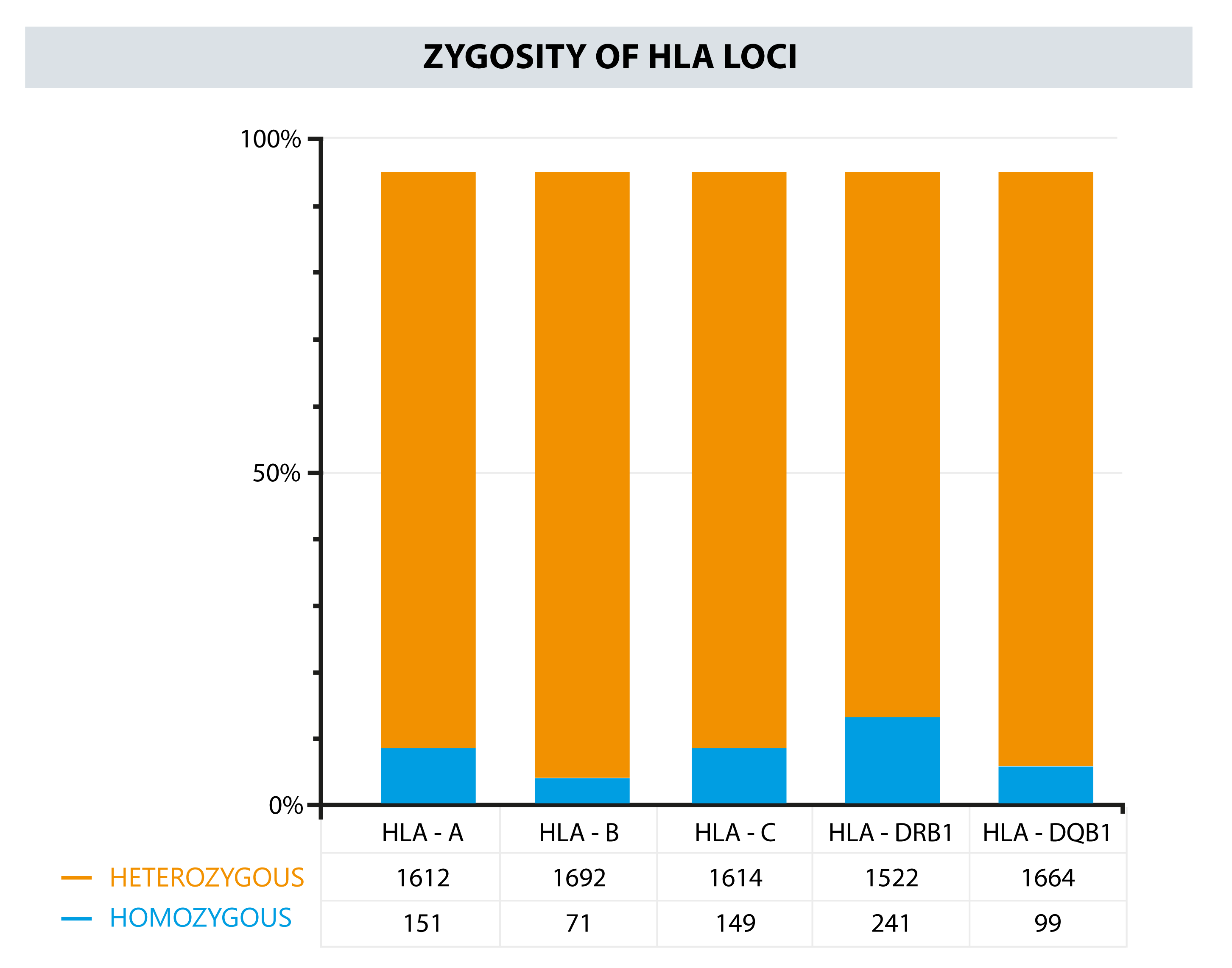


**Supplementary Figure 2.** Zygosity of the five HLA loci registered in the Colombian BM Donor Registry.

## Supplementary Tables

**Supplementary Table 1.** Count of the 1749 Colombian donors registered in the Colombian BM Donor Registry by the department of origin.

| **DEPARTMENT** | **NUMBER OF PEOPLE** |
| --- | --- |
| Bogota | 982 |
| Antioquia | 168 |
| Valle del Cauca | 127 |
| Atlántico | 117 |
| Cundinamarca | 72 |
| Boyacá | 41 |
| Santander | 35 |
| Tolima | 35 |
| Caldas | 17 |
| Bolivar | 16 |
| Magdalena | 16 |
| Narino | 16 |
| North of Santander | 16 |
| Huila | 13 |
| Cease | 12 |
| Risaralda | 11 |
| Goal | 10 |
| Cauca | 9 |
| La Guajira | 8 |
| Quindío | 6 |
| Caquetá | 4 |
| Casanare | 4 |
| Arauca | 3 |
| Cordoba | 3 |
| Sucre | 3 |
| Putumayo | 2 |
| Chocó | 1 |
| San Andrés | 1 |
| Vichada | 1 |
| Amazon | 0 |
| Vaupes | 0 |
| Guainia | 0 |
| Guaviare | 0 |

**Supplementary Table 2.** Complete table with allele frequencies for the 5 HLA loci.

| **HLA-A** | **Frequency** | **HLA-B** | **Frequency** | **HLA-C** | **Frequency** | **HLA-DQB1** | **Frequency** | **HLA-DRB1** | **Frequency** |
| --- | --- | --- | --- | --- | --- | --- | --- | --- | --- |
| A*24:02g | 0.208735 | B*35:43g | 0.076858 | C*04:01g | 0.153999 | DQB1*03:02g | 0.209586 | DRB1*04:07g | 0.110323 |
| A*02:01g | 0.161089 | B*40:02g | 0.071753 | C*01:02g | 0.104935 | DQB1*03:01g | 0.177822 | DRB1*07:01g | 0.097845 |
| A*01:01g | 0.070618 | B*44:03g | 0.060692 | C*07:02g | 0.104368 | DQB1*02:01g | 0.160522 | DRB1*08:02g | 0.067215 |
| A*03:01g | 0.068349 | B*51:01g | 0.058990 | C*07:01g | 0.089336 | DQB1*05:01g | 0.119115 | DRB1*03:01g | 0.065797 |
| A*29:02g | 0.052184 | B*07:02g | 0.053602 | C*03:04g | 0.080545 | DQB1*04:02g | 0.111174 | DRB1*15:01g | 0.063812 |
| A*68:01g | 0.049915 | B*35:01g | 0.052467 | C*08:02g | 0.060692 | DQB1*06:02g | 0.070335 | DRB1*13:01g | 0.051333 |
| A*11:01g | 0.043676 | B*14:02g | 0.051049 | C*16:01g | 0.053602 | DQB1*06:03g | 0.051333 | DRB1*01:01g | 0.044526 |
| A*23:01g | 0.032048 | B*18:01g | 0.038571 | C*06:02g | 0.049631 | DQB1*06:04g | 0.026375 | DRB1*04:05g | 0.043392 |
| A*31:01g | 0.031764 | B*08:01g | 0.035167 | C*05:01g | 0.047646 | DQB1*03:03g | 0.024957 | DRB1*14:02g | 0.042258 |
| A*26:01g | 0.031764 | B*44:02g | 0.030913 | C*12:03g | 0.040272 | DQB1*05:03g | 0.017016 | DRB1*13:02g | 0.039705 |
| A*02:22g | 0.026943 | B*38:01g | 0.025241 | C*15:02g | 0.039138 | DQB1*06:09g | 0.011344 | DRB1*01:02g | 0.039138 |
| A*33:01g | 0.021271 | B*39:05g | 0.024957 | C*02:02g | 0.038571 | DQB1*05:02g | 0.009926 | DRB1*11:01g | 0.036585 |
| A*68:02g | 0.019853 | B*49:01g | 0.024390 | C*03:05g | 0.031764 | DQB1*06:01g | 0.006807 | DRB1*04:04g | 0.036302 |
| A*32:01g | 0.019285 | B*35:12g | 0.023256 | C*03:03g | 0.019002 | DQB1*03:04g | 0.001418 | DRB1*16:02g | 0.031197 |
| A*30:02g | 0.019002 | B*15:01g | 0.018434 | C*17:01g | 0.016733 | DQB1*03:05g | 0.000567 | DRB1*11:04g | 0.019285 |
| A*02:13 | 0.017584 | B*57:01g | 0.017867 | C*08:01g | 0.013330 | DQB1*03:14 | 0.000567 | DRB1*10:01g | 0.016733 |
| A*24:03g | 0.016449 | B*53:01g | 0.017016 | C*14:02g | 0.012762 | DQB1*03:72 | 0.000284 | DRB1*04:11 | 0.016449 |
| A*02:05g | 0.014748 | B*27:05g | 0.016449 | C*12:02g | 0.007374 | DQB1*05:04g | 0.000284 | DRB1*14:01g | 0.016166 |
| A*30:01g | 0.012195 | B*50:01g | 0.016449 | C*16:02g | 0.005105 | DQB1*06:08 | 0.000284 | DRB1*01:03g | 0.013897 |
| A*24:14 | 0.011628 | B*48:01g | 0.015598 | C*15:05g | 0.004821 | DQB1*06:11 | 0.000284 | DRB1*04:02g | 0.013897 |
| A*02:11g | 0.010210 | B*40:01g | 0.012762 | C*07:04g | 0.003403 |  |  | DRB1*03:02g | 0.012762 |
| A*25:01g | 0.008508 | B*45:01g | 0.012195 | C*08:03g | 0.003120 |  |  | DRB1*13:03g | 0.012479 |
| A*33:03g | 0.007090 | B*52:01g | 0.011628 | C*18:01g | 0.003120 |  |  | DRB1*09:01g | 0.011912 |
| A*74:01g | 0.005389 | B*14:01g | 0.011344 | C*03:02g | 0.002552 |  |  | DRB1*04:03g | 0.011061 |
| A*66:01g | 0.004254 | B*39:11 | 0.011061 | C*16:04 | 0.002269 |  |  | DRB1*11:02g | 0.010777 |
| A*34:02g | 0.004254 | B*35:03g | 0.010493 | C*01:10 | 0.001702 |  |  | DRB1*04:01g | 0.008792 |
| A*03:02g | 0.004254 | B*55:01g | 0.010210 | C*03:06 | 0.001702 |  |  | DRB1*08:01g | 0.008792 |
| A*02:02g | 0.004254 | B*35:31 | 0.009643 | C*15:04g | 0.001702 |  |  | DRB1*15:03g | 0.007941 |
| A*02:04g | 0.003120 | B*58:01g | 0.009643 | C*08:08 | 0.001134 |  |  | DRB1*12:01g | 0.007657 |
| A*30:04g | 0.002552 | B*13:02g | 0.009075 | C*08:04 | 0.000851 |  |  | DRB1*08:04g | 0.006807 |
| A*69:01 | 0.002269 | B*15:03g | 0.009075 | C*14:03g | 0.000851 |  |  | DRB1*15:02g | 0.005956 |
| A*29:01g | 0.001985 | B*41:01g | 0.008225 | C*04:07 | 0.000567 |  |  | DRB1*16:01g | 0.005672 |
| A*02:17 | 0.001985 | B*35:02g | 0.007941 | C*05:04 | 0.000567 |  |  | DRB1*04:10g | 0.003971 |
| A*36:01g | 0.001702 | B*35:08g | 0.007657 | C*05:09 | 0.000567 |  |  | DRB1*07:11 | 0.003403 |
| A*80:01g | 0.001418 | B*35:20 | 0.007090 | C*07:326 | 0.000567 |  |  | DRB1*04:08g | 0.002552 |
| A*02:06g | 0.001418 | B*39:06g | 0.006523 | C*02:14g | 0.000284 |  |  | DRB1*11:03g | 0.002552 |
| A*26:08 | 0.000851 | B*40:04 | 0.006523 | C*04:113 | 0.000284 |  |  | DRB1*13:04 | 0.002552 |
| A*66:02g | 0.000567 | B*15:17g | 0.006239 | C*04:244 | 0.000284 |  |  | DRB1*13:05 | 0.002269 |
| A*31:02 | 0.000567 | B*35:30 | 0.006239 | C*06:08 | 0.000284 |  |  | DRB1*08:03g | 0.001134 |
| A*24:53 | 0.000567 | B*35:04 | 0.005105 | C*07:108 | 0.000284 |  |  | DRB1*08:06 | 0.001134 |
| A*24:15 | 0.000567 | B*35:05g | 0.005105 | C*07:172 | 0.000284 |  |  | DRB1*14:06 | 0.001134 |
| A*68:55 | 0.000284 | B*37:01g | 0.004821 |  |  |  |  | DRB1*08:07 | 0.000851 |
| A*68:05 | 0.000284 | B*39:01g | 0.004538 |  |  |  |  | DRB1*04:06 | 0.000567 |
| A*31:15 | 0.000284 | B*41:02g | 0.004538 |  |  |  |  | DRB1*14:07 | 0.000567 |
| A*30:172 | 0.000284 | B*58:02g | 0.004254 |  |  |  |  | DRB1*04:17 | 0.000284 |
| A*30:10 | 0.000284 | B*15:16g | 0.003971 |  |  |  |  | DRB1*11:14 | 0.000284 |
| A*30:09 | 0.000284 | B*15:10g | 0.003687 |  |  |  |  | DRB1*12:02g | 0.000284 |
| A*24:07g | 0.000284 | B*39:08 | 0.003403 |  |  |  |  |  |  |
| A*02:64 | 0.000284 | B*07:05g | 0.003120 |  |  |  |  |  |  |
| A*02:07g | 0.000284 | B*35:10 | 0.003120 |  |  |  |  |  |  |
| A*01:03g | 0.000284 | B*42:01g | 0.003120 |  |  |  |  |  |  |
| A*01:02 | 0.000284 | B*50:02 | 0.002836 |  |  |  |  |  |  |
|  |  | B*42:02 | 0.002552 |  |  |  |  |  |  |
|  |  | B*56:01g | 0.002552 |  |  |  |  |  |  |
|  |  | B*57:03g | 0.002552 |  |  |  |  |  |  |
|  |  | B*35:11 | 0.001985 |  |  |  |  |  |  |
|  |  | B*35:49 | 0.001985 |  |  |  |  |  |  |
|  |  | B*51:10 | 0.001985 |  |  |  |  |  |  |
|  |  | B*27:02g | 0.001702 |  |  |  |  |  |  |
|  |  | B*39:09g | 0.001702 |  |  |  |  |  |  |
|  |  | B*48:02 | 0.001702 |  |  |  |  |  |  |
|  |  | B*51:08g | 0.001702 |  |  |  |  |  |  |
|  |  | B*15:04 | 0.001418 |  |  |  |  |  |  |
|  |  | B*57:02 | 0.001418 |  |  |  |  |  |  |
|  |  | B*40:11 | 0.001134 |  |  |  |  |  |  |
|  |  | B*47:01g | 0.001134 |  |  |  |  |  |  |
|  |  | B*81:01g | 0.001134 |  |  |  |  |  |  |
|  |  | B*35:22 | 0.000851 |  |  |  |  |  |  |
|  |  | B*35:44 | 0.000851 |  |  |  |  |  |  |
|  |  | B*39:10g | 0.000851 |  |  |  |  |  |  |
|  |  | B*40:03g | 0.000851 |  |  |  |  |  |  |
|  |  | B*40:06g | 0.000851 |  |  |  |  |  |  |
|  |  | B*51:07 | 0.000851 |  |  |  |  |  |  |
|  |  | B*07:26 | 0.000567 |  |  |  |  |  |  |
|  |  | B*15:05 | 0.000567 |  |  |  |  |  |  |
|  |  | B*15:18g | 0.000567 |  |  |  |  |  |  |
|  |  | B*15:31 | 0.000567 |  |  |  |  |  |  |
|  |  | B*15:39g | 0.000567 |  |  |  |  |  |  |
|  |  | B*18:147 | 0.000567 |  |  |  |  |  |  |
|  |  | B*39:02g | 0.000567 |  |  |  |  |  |  |
|  |  | B*39:03g | 0.000567 |  |  |  |  |  |  |
|  |  | B*39:13 | 0.000567 |  |  |  |  |  |  |
|  |  | B*07:09 | 0.000284 |  |  |  |  |  |  |
|  |  | B*14:03 | 0.000284 |  |  |  |  |  |  |
|  |  | B*15:02g | 0.000284 |  |  |  |  |  |  |
|  |  | B*15:07g | 0.000284 |  |  |  |  |  |  |
|  |  | B*15:09 | 0.000284 |  |  |  |  |  |  |
|  |  | B*15:110 | 0.000284 |  |  |  |  |  |  |
|  |  | B*15:20g | 0.000284 |  |  |  |  |  |  |
|  |  | B*18:03 | 0.000284 |  |  |  |  |  |  |
|  |  | B*18:07 | 0.000284 |  |  |  |  |  |  |
|  |  | B*18:26 | 0.000284 |  |  |  |  |  |  |
|  |  | B*27:03 | 0.000284 |  |  |  |  |  |  |
|  |  | B*27:08 | 0.000284 |  |  |  |  |  |  |
|  |  | B*27:12 | 0.000284 |  |  |  |  |  |  |
|  |  | B*35:117 | 0.000284 |  |  |  |  |  |  |
|  |  | B*35:475 | 0.000284 |  |  |  |  |  |  |
|  |  | B*35:476 | 0.000284 |  |  |  |  |  |  |
|  |  | B*35:68 | 0.000284 |  |  |  |  |  |  |
|  |  | B*39:07 | 0.000284 |  |  |  |  |  |  |
|  |  | B*39:22 | 0.000284 |  |  |  |  |  |  |
|  |  | B*39:24 | 0.000284 |  |  |  |  |  |  |
|  |  | B*40:05 | 0.000284 |  |  |  |  |  |  |
|  |  | B*44:04 | 0.000284 |  |  |  |  |  |  |
|  |  | B*44:05g | 0.000284 |  |  |  |  |  |  |
|  |  | B*44:06 | 0.000284 |  |  |  |  |  |  |
|  |  | B*44:10 | 0.000284 |  |  |  |  |  |  |
|  |  | B*55:11 | 0.000284 |  |  |  |  |  |  |
|  |  | B*73:01g | 0.000284 |  |  |  |  |  |  |
|  |  | B*78:01 | 0.000284 |  |  |  |  |  |  |
|  |  | B*82:02 | 0.000284 |  |  |  |  |  |  |

**Supplementary Table 3.** Comparison table of the 11 most common HLA alleles and their frequencies between the 1347 women and 416 men registered in the Colombian BM Donor Registry.

| *Allele* | *Female (%)* | *Male (%)* | *Ratio (male/female)* |
| --- | --- | --- | --- |
| A*24:02g | 0,207 | 0,213 | 1,025 |
| A*02:01g | 0,158 | 0,172 | 1,089 |
| A*01:01g | 0,069 | 0,075 | 1,074 |
| A*03:01g | 0,073 | 0,052 | 0,703 |
| A*29:02g | 0,053 | 0,050 | 0,958 |
| A*68:01g | 0,050 | 0,049 | 0,983 |
| A*11:01g | 0,043 | 0,044 | 1,024 |
| A*23:01g | 0,034 | 0,026 | 0,783 |
| A*26:01g | 0,033 | 0,026 | 0,792 |
| A*31:01g | 0,032 | 0,030 | 0,930 |
| A*02:22g | 0,027 | 0,028 | 1,034 |
| *Allele* | ***Female (%)*** | ***Male (%)*** | ***Ratio (male/female)*** |
| B*35:43g | 0,075 | 0,082 | 1,085 |
| B*40:02g | 0,072 | 0,070 | 0,963 |
| B*44:03g | 0,063 | 0,052 | 0,814 |
| B*51:01g | 0,057 | 0,065 | 1,135 |
| B*07:02g | 0,056 | 0,046 | 0,815 |
| B*35:01g | 0,053 | 0,050 | 0,951 |
| B*14:02g | 0,052 | 0,049 | 0,955 |
| B*18:01g | 0,040 | 0,032 | 0,802 |
| B*08:01g | 0,034 | 0,040 | 1,174 |
| B*44:02g | 0,032 | 0,029 | 0,914 |
| B*38:01g | 0,025 | 0,025 | 1,000 |
| *Allele* | ***Female (%)*** | ***Male (%)*** | ***Ratio (male/female)*** |
| C*04:01g | 0,156 | 0,148 | 0,948 |
| C*01:02g | 0,104 | 0,107 | 1,026 |
| C*07:02g | 0,105 | 0,102 | 0,973 |
| C*07:01g | 0,085 | 0,103 | 1,216 |
| C*03:04g | 0,079 | 0,084 | 1,059 |
| C*08:02g | 0,063 | 0,053 | 0,838 |
| C*16:01g | 0,055 | 0,050 | 0,925 |
| C*06:02g | 0,048 | 0,056 | 1,189 |
| C*05:01g | 0,049 | 0,043 | 0,883 |
| C*12:03g | 0,042 | 0,035 | 0,831 |
| C*15:02g | 0,037 | 0,044 | 1,186 |
| *Allele* | ***Female (%)*** | ***Male (%)*** | ***Ratio (male/female)*** |
| DQB1*03:02g | 0,211 | 0,206 | 0,975 |
| DQB1*03:01g | 0,179 | 0,173 | 0,965 |
| DQB1*02:01g | 0,158 | 0,168 | 1,064 |
| DQB1*05:01g | 0,120 | 0,118 | 0,985 |
| DQB1*04:02g | 0,107 | 0,124 | 1,154 |
| DQB1*06:02g | 0,074 | 0,058 | 0,777 |
| DQB1*06:03g | 0,049 | 0,059 | 1,202 |
| DQB1*06:04g | 0,026 | 0,026 | 1,003 |
| DQB1*03:03g | 0,026 | 0,023 | 0,892 |
| DQB1*05:03g | 0,019 | 0,012 | 0,648 |
| DQB1*06:09g | 0,012 | 0,011 | 0,940 |
| *Allele* | ***Female (%)*** | ***Male (%)*** | ***Ratio (male/female)*** |
| DRB1*04:07g | 0,111 | 0,108 | 0,975 |
| DRB1*07:01g | 0,098 | 0,096 | 0,978 |
| DRB1*08:02g | 0,063 | 0,079 | 1,250 |
| DRB1*03:01g | 0,063 | 0,073 | 1,155 |
| DRB1*15:01g | 0,065 | 0,060 | 0,925 |
| DRB1*13:01g | 0,049 | 0,060 | 1,236 |
| DRB1*01:01g | 0,045 | 0,043 | 0,963 |
| DRB1*04:05g | 0,042 | 0,049 | 1,185 |
| DRB1*14:02g | 0,039 | 0,053 | 1,357 |
| DRB1*13:02g | 0,040 | 0,038 | 0,959 |
| DRB1*01:02g | 0,040 | 0,036 | 0,899 |

**Supplementary Table 4.** Complete list of HLA A~B~C~DQB1~DRB1 haplotypes with a frequency greater than 0.1%.

| **Haplotype** | **Frequency** |
| --- | --- |
| A*24:02g~B*35:43g~C*01:02g~DQB1*03:02g~DRB1*04:07g | 0.03335465114724 |
| A*29:02g~B*44:03g~C*16:01g~DQB1*02:01g~DRB1*07:01g | 0.02035363713968 |
| A*01:01g~B*08:01g~C*07:01g~DQB1*02:01g~DRB1*03:01g | 0.01828632955033 |
| A*33:01g~B*14:02g~C*08:02g~DQB1*05:01g~DRB1*01:02g | 0.01134432259299 |
| A*03:01g~B*07:02g~C*07:02g~DQB1*06:02g~DRB1*15:01g | 0.01047698060923 |
| A*24:02g~B*40:02g~C*03:05g~DQB1*03:02g~DRB1*04:07g | 0.00908537068506 |
| A*24:02g~B*35:43g~C*01:02g~DQB1*04:02g~DRB1*08:02g | 0.00857410476289 |
| A*24:02g~B*35:12g~C*04:01g~DQB1*03:01g~DRB1*16:02g | 0.00844378222345 |
| A*03:01g~B*35:01g~C*04:01g~DQB1*05:01g~DRB1*01:01g | 0.00780892490033 |
| A*24:02g~B*07:02g~C*07:02g~DQB1*06:02g~DRB1*15:01g | 0.00752087496157 |
| A*30:02g~B*18:01g~C*05:01g~DQB1*02:01g~DRB1*03:01g | 0.00708663754494 |
| A*02:01g~B*07:02g~C*07:02g~DQB1*05:01g~DRB1*01:03g | 0.00651679051341 |
| A*24:02g~B*35:12g~C*04:01g~DQB1*03:02g~DRB1*04:07g | 0.00575807313789 |
| A*02:01g~B*18:01g~C*05:01g~DQB1*02:01g~DRB1*03:01g | 0.00535989784130 |
| A*02:01g~B*07:02g~C*07:02g~DQB1*06:02g~DRB1*15:01g | 0.00524695311905 |
| A*02:13~B*51:01g~C*15:02g~DQB1*03:02g~DRB1*04:04g | 0.00506661886177 |
| A*02:01g~B*44:02g~C*05:01g~DQB1*06:03g~DRB1*13:01g | 0.00499120193423 |
| A*24:02g~B*35:43g~C*01:02g~DQB1*03:01g~DRB1*14:02g | 0.00481164279596 |
| A*02:01g~B*38:01g~C*12:03g~DQB1*06:03g~DRB1*13:01g | 0.00445827224912 |
| A*02:01g~B*44:02g~C*05:01g~DQB1*05:01g~DRB1*01:01g | 0.00444640693181 |
| A*02:01g~B*14:02g~C*08:02g~DQB1*05:01g~DRB1*01:02g | 0.00438840121255 |
| A*23:01g~B*44:03g~C*04:01g~DQB1*02:01g~DRB1*07:01g | 0.00397051290767 |
| A*30:01g~B*13:02g~C*06:02g~DQB1*02:01g~DRB1*07:01g | 0.00397051290755 |
| A*11:01g~B*27:05g~C*01:02g~DQB1*05:01g~DRB1*01:01g | 0.00362701204616 |
| A*24:02g~B*48:01g~C*08:01g~DQB1*04:02g~DRB1*08:02g | 0.00356986423994 |
| A*02:05g~B*50:01g~C*06:02g~DQB1*02:01g~DRB1*07:01g | 0.00340329677790 |
| A*26:01g~B*38:01g~C*12:03g~DQB1*06:03g~DRB1*13:01g | 0.00340095986509 |
| A*02:22g~B*35:01g~C*04:01g~DQB1*04:02g~DRB1*04:05g | 0.00340060455684 |
| A*02:11g~B*40:04~C*03:04g~DQB1*03:01g~DRB1*14:02g | 0.00339654773675 |
| A*24:14~B*40:02g~C*03:04g~DQB1*03:02g~DRB1*04:07g | 0.00337118313678 |
| A*31:01g~B*35:43g~C*01:02g~DQB1*03:02g~DRB1*04:07g | 0.00332589098814 |
| A*24:02g~B*40:02g~C*03:05g~DQB1*04:02g~DRB1*08:02g | 0.00331486336376 |
| A*24:02g~B*40:02g~C*03:05g~DQB1*03:01g~DRB1*14:02g | 0.00324978590065 |
| A*24:02g~B*45:01g~C*16:01g~DQB1*05:01g~DRB1*10:01g | 0.00311734652241 |
| A*02:13~B*35:43g~C*01:02g~DQB1*03:02g~DRB1*04:07g | 0.00311733732998 |
| A*11:01g~B*35:01g~C*04:01g~DQB1*05:03g~DRB1*14:01g | 0.00311655388889 |
| A*68:02g~B*14:02g~C*08:02g~DQB1*05:01g~DRB1*01:02g | 0.00310089268023 |
| A*11:01g~B*35:01g~C*04:01g~DQB1*05:01g~DRB1*01:03g | 0.00283191355770 |
| A*02:01g~B*13:02g~C*06:02g~DQB1*02:01g~DRB1*07:01g | 0.00278379002351 |
| A*68:01g~B*39:05g~C*07:02g~DQB1*03:02g~DRB1*04:07g | 0.00273534066577 |
| A*24:02g~B*14:02g~C*08:02g~DQB1*02:01g~DRB1*07:11 | 0.00265342647346 |
| A*02:01g~B*35:43g~C*01:02g~DQB1*03:02g~DRB1*04:07g | 0.00260601280792 |
| A*24:02g~B*40:02g~C*03:04g~DQB1*03:02g~DRB1*04:04g | 0.00256943304538 |
| A*24:03g~B*35:43g~C*01:02g~DQB1*03:02g~DRB1*04:07g | 0.00256708545531 |
| A*26:01g~B*38:01g~C*12:03g~DQB1*03:02g~DRB1*04:02g | 0.00255247260408 |
| A*01:01g~B*57:01g~C*06:02g~DQB1*03:03g~DRB1*07:01g | 0.00255247258342 |
| A*26:01g~B*38:01g~C*12:03g~DQB1*05:03g~DRB1*14:01g | 0.00255247258342 |
| A*02:01g~B*51:01g~C*15:02g~DQB1*03:01g~DRB1*11:01g | 0.00255247258342 |
| A*68:01g~B*35:31~C*03:04g~DQB1*03:02g~DRB1*04:07g | 0.00255247258342 |
| A*02:01g~B*40:02g~C*04:01g~DQB1*04:02g~DRB1*08:02g | 0.00255111256898 |
| A*01:01g~B*57:01g~C*07:01g~DQB1*03:03g~DRB1*07:01g | 0.00254138241018 |
| A*68:01g~B*35:20~C*04:01g~DQB1*04:02g~DRB1*08:02g | 0.00253986190473 |
| A*29:02g~B*07:02g~C*07:02g~DQB1*06:02g~DRB1*15:01g | 0.00244655781999 |
| A*11:01g~B*35:01g~C*04:01g~DQB1*05:01g~DRB1*01:01g | 0.00239706782231 |
| A*68:01g~B*40:02g~C*03:05g~DQB1*04:02g~DRB1*08:02g | 0.00227665559693 |
| A*25:01g~B*18:01g~C*12:03g~DQB1*06:02g~DRB1*15:01g | 0.00226886451860 |
| A*11:01g~B*40:01g~C*03:04g~DQB1*03:02g~DRB1*04:04g | 0.00226886451860 |
| A*24:02g~B*35:02g~C*04:01g~DQB1*03:01g~DRB1*11:04g | 0.00226417875297 |
| A*02:01g~B*15:17g~C*07:01g~DQB1*06:04g~DRB1*13:02g | 0.00222598605998 |
| A*02:01g~B*50:01g~C*06:02g~DQB1*02:01g~DRB1*03:01g | 0.00221566814651 |
| A*02:01g~B*44:03g~C*16:01g~DQB1*02:01g~DRB1*07:01g | 0.00220918757132 |
| A*02:22g~B*40:02g~C*03:04g~DQB1*04:02g~DRB1*08:02g | 0.00215693911685 |
| A*24:14~B*40:02g~C*03:04g~DQB1*04:02g~DRB1*08:02g | 0.00214471200468 |
| A*01:01g~B*52:01g~C*12:02g~DQB1*06:01g~DRB1*15:02g | 0.00198525645377 |
| A*24:02g~B*18:01g~C*12:03g~DQB1*03:01g~DRB1*11:04g | 0.00198525645377 |
| A*24:02g~B*35:31~C*03:05g~DQB1*03:02g~DRB1*04:07g | 0.00198525645377 |
| A*02:01g~B*15:01g~C*03:04g~DQB1*03:02g~DRB1*04:01g | 0.00198522273963 |
| A*24:02g~B*35:30~C*03:04g~DQB1*03:01g~DRB1*16:02g | 0.00198369365739 |
| A*24:02g~B*39:05g~C*07:02g~DQB1*04:02g~DRB1*08:02g | 0.00197233361481 |
| A*68:01g~B*35:31~C*03:04g~DQB1*04:02g~DRB1*08:02g | 0.00196719059550 |
| A*02:01g~B*50:01g~C*07:01g~DQB1*03:03g~DRB1*07:01g | 0.00196356189952 |
| A*29:02g~B*57:01g~C*07:01g~DQB1*05:01g~DRB1*01:01g | 0.00196331399549 |
| A*01:01g~B*44:03g~C*16:01g~DQB1*02:01g~DRB1*07:01g | 0.00195448540552 |
| A*24:02g~B*39:05g~C*07:02g~DQB1*03:01g~DRB1*14:02g | 0.00193638600402 |
| A*02:01g~B*39:11~C*07:02g~DQB1*04:02g~DRB1*04:10g | 0.00192308287463 |
| A*29:02g~B*44:03g~C*16:01g~DQB1*06:02g~DRB1*15:01g | 0.00187422094951 |
| A*26:01g~B*14:01g~C*08:02g~DQB1*02:01g~DRB1*07:01g | 0.00187422094951 |
| A*02:01g~B*39:11~C*07:02g~DQB1*03:01g~DRB1*14:02g | 0.00178573907751 |
| A*02:22g~B*39:05g~C*07:02g~DQB1*04:02g~DRB1*08:02g | 0.00176522345716 |
| A*29:02g~B*44:03g~C*16:01g~DQB1*06:03g~DRB1*13:01g | 0.00173486423801 |
| A*24:02g~B*35:02g~C*04:01g~DQB1*02:01g~DRB1*03:01g | 0.00170494366361 |
| A*68:01g~B*45:01g~C*06:02g~DQB1*03:02g~DRB1*04:05g | 0.00170164838895 |
| A*02:01g~B*51:01g~C*14:02g~DQB1*04:02g~DRB1*08:01g | 0.00170164838895 |
| A*31:01g~B*40:01g~C*03:04g~DQB1*03:02g~DRB1*04:04g | 0.00170164838895 |
| A*03:02g~B*44:02g~C*16:04~DQB1*03:02g~DRB1*04:02g | 0.00170164838895 |
| A*30:02g~B*27:05g~C*01:02g~DQB1*06:03g~DRB1*13:01g | 0.00170164838895 |
| A*02:01g~B*51:01g~C*14:02g~DQB1*06:03g~DRB1*13:01g | 0.00170164838895 |
| A*32:01g~B*14:02g~C*08:02g~DQB1*05:01g~DRB1*01:02g | 0.00170164838895 |
| A*02:01g~B*49:01g~C*07:01g~DQB1*06:04g~DRB1*13:02g | 0.00170164838894 |
| A*24:02g~B*40:02g~C*03:04g~DQB1*03:01g~DRB1*03:02g | 0.00170164838891 |
| A*02:01g~B*48:01g~C*08:01g~DQB1*04:02g~DRB1*04:05g | 0.00170153432794 |
| A*11:01g~B*53:01g~C*04:01g~DQB1*06:04g~DRB1*13:02g | 0.00169860262044 |
| A*02:05g~B*49:01g~C*07:01g~DQB1*03:02g~DRB1*04:05g | 0.00169363423031 |
| A*02:01g~B*08:01g~C*07:01g~DQB1*02:01g~DRB1*03:01g | 0.00168280640626 |
| A*68:01g~B*39:05g~C*07:02g~DQB1*04:02g~DRB1*08:02g | 0.00166705483965 |
| A*24:02g~B*39:05g~C*07:02g~DQB1*03:02g~DRB1*04:07g | 0.00162137575676 |
| A*02:01g~B*39:11~C*07:02g~DQB1*04:02g~DRB1*08:02g | 0.00154368163425 |
| A*68:01g~B*39:11~C*07:02g~DQB1*03:01g~DRB1*14:02g | 0.00148540041527 |
| A*24:02g~B*40:02g~C*03:04g~DQB1*03:02g~DRB1*04:07g | 0.00145696506014 |
| A*02:01g~B*35:43g~C*01:02g~DQB1*04:02g~DRB1*08:02g | 0.00143767573697 |
| A*24:02g~B*35:20~C*04:01g~DQB1*04:02g~DRB1*08:02g | 0.00142285992449 |
| A*33:01g~B*14:02g~C*08:02g~DQB1*03:02g~DRB1*04:04g | 0.00141804032412 |
| A*31:01g~B*39:05g~C*07:02g~DQB1*03:02g~DRB1*04:11 | 0.00141804032412 |
| A*26:01g~B*49:01g~C*15:04g~DQB1*03:01g~DRB1*11:01g | 0.00141804032412 |
| A*01:01g~B*41:01g~C*17:01g~DQB1*05:01g~DRB1*10:01g | 0.00141804032412 |
| A*03:01g~B*40:01g~C*03:04g~DQB1*03:02g~DRB1*04:03g | 0.00141804032412 |
| A*01:01g~B*35:02g~C*04:01g~DQB1*06:03g~DRB1*11:04g | 0.00141804032412 |
| A*11:01g~B*41:01g~C*17:01g~DQB1*02:01g~DRB1*04:05g | 0.00141804032412 |
| A*02:05g~B*14:02g~C*08:02g~DQB1*02:01g~DRB1*03:01g | 0.00141804032412 |
| A*02:01g~B*40:01g~C*03:04g~DQB1*06:02g~DRB1*15:01g | 0.00141804032412 |
| A*24:02g~B*14:01g~C*08:02g~DQB1*02:01g~DRB1*07:01g | 0.00141804032412 |
| A*24:02g~B*39:06g~C*07:02g~DQB1*06:02g~DRB1*15:01g | 0.00141804032412 |
| A*11:01g~B*55:01g~C*03:03g~DQB1*05:03g~DRB1*14:01g | 0.00141804032412 |
| A*24:03g~B*35:04~C*04:01g~DQB1*03:02g~DRB1*04:07g | 0.00141804032412 |
| A*33:01g~B*44:03g~C*04:01g~DQB1*02:01g~DRB1*07:01g | 0.00141804032412 |
| A*02:01g~B*49:01g~C*07:01g~DQB1*03:02g~DRB1*04:05g | 0.00141803687773 |
| A*24:03g~B*35:12g~C*04:01g~DQB1*03:01g~DRB1*16:02g | 0.00141802596816 |
| A*03:01g~B*15:16g~C*14:02g~DQB1*03:02g~DRB1*04:05g | 0.00141799193604 |
| A*68:01g~B*49:01g~C*07:01g~DQB1*05:01g~DRB1*10:01g | 0.00141374435297 |
| A*02:22g~B*40:02g~C*03:04g~DQB1*03:02g~DRB1*04:04g | 0.00137814124038 |
| A*24:02g~B*14:02g~C*08:02g~DQB1*05:01g~DRB1*01:02g | 0.00134078915073 |
| A*24:02g~B*44:03g~C*16:01g~DQB1*02:01g~DRB1*07:01g | 0.00133451395938 |
| A*24:02g~B*35:12g~C*04:01g~DQB1*03:01g~DRB1*14:02g | 0.00131926294343 |
| A*11:01g~B*51:01g~C*15:02g~DQB1*06:02g~DRB1*15:01g | 0.00127634707905 |
| A*68:01g~B*35:43g~C*01:02g~DQB1*03:02g~DRB1*04:07g | 0.00126903939983 |
| A*02:01g~B*55:01g~C*03:03g~DQB1*02:01g~DRB1*07:01g | 0.00124645196762 |
| A*03:01g~B*14:02g~C*08:02g~DQB1*05:01g~DRB1*01:02g | 0.00123484333674 |
| A*01:01g~B*07:02g~C*07:02g~DQB1*06:02g~DRB1*15:01g | 0.00122664035774 |
| A*24:02g~B*35:43g~C*01:02g~DQB1*04:02g~DRB1*04:05g | 0.00120279961352 |
| A*03:01g~B*08:01g~C*07:01g~DQB1*02:01g~DRB1*03:01g | 0.00119613660779 |
| A*03:01g~B*51:01g~C*15:02g~DQB1*02:01g~DRB1*07:01g | 0.00118849228948 |
| A*03:01g~B*18:01g~C*05:01g~DQB1*02:01g~DRB1*03:01g | 0.00115648133591 |
| A*02:22g~B*35:43g~C*01:02g~DQB1*03:02g~DRB1*04:07g | 0.00115139272125 |
| A*01:01g~B*08:01g~C*07:01g~DQB1*06:03g~DRB1*13:01g | 0.00113925633790 |
| A*02:01g~B*44:02g~C*05:01g~DQB1*06:02g~DRB1*15:01g | 0.00113867295470 |
| A*29:02g~B*44:03g~C*16:01g~DQB1*05:03g~DRB1*14:01g | 0.00113756708218 |
| A*02:01g~B*51:01g~C*15:02g~DQB1*02:01g~DRB1*07:01g | 0.00113471935734 |
| A*24:02g~B*35:43g~C*01:02g~DQB1*03:01g~DRB1*03:02g | 0.00113443225933 |
| A*24:02g~B*40:02g~C*03:04g~DQB1*03:01g~DRB1*16:02g | 0.00113443225931 |
| A*68:01g~B*49:01g~C*03:04g~DQB1*06:04g~DRB1*13:02g | 0.00113443225931 |
| A*68:01g~B*39:11~C*07:02g~DQB1*03:01g~DRB1*16:02g | 0.00113443225930 |
| A*31:01g~B*40:02g~C*01:10~DQB1*04:02g~DRB1*08:02g | 0.00113443225930 |
| A*02:01g~B*51:01g~C*15:02g~DQB1*03:02g~DRB1*04:04g | 0.00113443225930 |
| A*31:01g~B*51:01g~C*15:02g~DQB1*06:04g~DRB1*13:02g | 0.00113443225930 |
| A*11:01g~B*52:01g~C*12:02g~DQB1*06:01g~DRB1*15:02g | 0.00113443225930 |
| A*69:01~B*55:01g~C*01:02g~DQB1*03:01g~DRB1*11:01g | 0.00113443225930 |
| A*30:01g~B*42:02~C*17:01g~DQB1*03:01g~DRB1*08:04g | 0.00113443225930 |
| A*26:01g~B*38:01g~C*12:03g~DQB1*03:01g~DRB1*11:03g | 0.00113443225930 |
| A*29:02g~B*07:02g~C*07:02g~DQB1*06:03g~DRB1*13:01g | 0.00113443225930 |
| A*30:04g~B*14:02g~C*08:02g~DQB1*03:01g~DRB1*12:01g | 0.00113443225930 |
| A*01:01g~B*50:01g~C*06:02g~DQB1*02:01g~DRB1*07:01g | 0.00113443225930 |
| A*03:01g~B*18:01g~C*07:01g~DQB1*04:02g~DRB1*04:05g | 0.00113443225930 |
| A*01:01g~B*15:17g~C*07:01g~DQB1*06:04g~DRB1*13:02g | 0.00113443225930 |
| A*30:02g~B*14:02g~C*08:02g~DQB1*06:02g~DRB1*15:03g | 0.00113443225930 |
| A*02:01g~B*18:01g~C*07:01g~DQB1*03:01g~DRB1*11:04g | 0.00113443225930 |
| A*01:01g~B*27:05g~C*02:02g~DQB1*03:01g~DRB1*11:02g | 0.00113443225930 |
| A*23:01g~B*15:03g~C*02:02g~DQB1*03:01g~DRB1*11:01g | 0.00113443225930 |
| A*32:01g~B*40:02g~C*02:02g~DQB1*06:03g~DRB1*13:01g | 0.00113443225930 |
| A*02:01g~B*51:01g~C*16:01g~DQB1*06:03g~DRB1*13:01g | 0.00113443225930 |
| A*68:02g~B*49:01g~C*07:01g~DQB1*05:01g~DRB1*10:01g | 0.00113443225930 |
| A*24:02g~B*40:02g~C*03:06~DQB1*03:01g~DRB1*14:02g | 0.00113443225930 |
| A*02:05g~B*58:01g~C*07:01g~DQB1*03:02g~DRB1*04:02g | 0.00113443225930 |
| A*11:01g~B*49:01g~C*07:01g~DQB1*03:02g~DRB1*04:05g | 0.00113443225930 |
| A*34:02g~B*14:01g~C*08:02g~DQB1*03:02g~DRB1*04:05g | 0.00113443225930 |
| A*33:03g~B*14:02g~C*08:02g~DQB1*06:03g~DRB1*13:01g | 0.00113443225930 |
| A*02:22g~B*35:01g~C*04:01g~DQB1*04:02g~DRB1*08:02g | 0.00113443225930 |
| A*24:02g~B*35:43g~C*08:08~DQB1*03:02g~DRB1*04:07g | 0.00113443225930 |
| A*23:01g~B*07:02g~C*07:02g~DQB1*06:02g~DRB1*15:03g | 0.00113443225930 |
| A*24:03g~B*35:04~C*04:01g~DQB1*03:01g~DRB1*14:02g | 0.00113443225930 |
| A*24:02g~B*39:05g~C*07:02g~DQB1*04:02g~DRB1*04:05g | 0.00113443225930 |
| A*23:01g~B*50:02~C*04:01g~DQB1*05:03g~DRB1*14:01g | 0.00113443225930 |
| A*24:02g~B*48:01g~C*01:02g~DQB1*04:02g~DRB1*08:02g | 0.00113443225930 |
| A*33:03g~B*35:08g~C*04:01g~DQB1*05:01g~DRB1*01:01g | 0.00113443225930 |
| A*02:01g~B*07:02g~C*07:02g~DQB1*05:01g~DRB1*01:01g | 0.00113443225632 |
| A*24:02g~B*15:01g~C*03:03g~DQB1*06:03g~DRB1*13:01g | 0.00113443225370 |
| A*68:02g~B*53:01g~C*04:01g~DQB1*06:04g~DRB1*13:02g | 0.00113276431359 |
| A*68:01g~B*35:01g~C*03:04g~DQB1*04:02g~DRB1*08:02g | 0.00113172847212 |
| A*68:02g~B*53:01g~C*04:01g~DQB1*05:01g~DRB1*01:02g | 0.00113149899973 |
| A*01:01g~B*41:02g~C*17:01g~DQB1*03:01g~DRB1*13:03g | 0.00112980405229 |
| A*02:01g~B*40:02g~C*03:05g~DQB1*03:01g~DRB1*16:02g | 0.00112800957419 |
| A*23:01g~B*44:03g~C*04:01g~DQB1*02:01g~DRB1*03:01g | 0.00111833154730 |
| A*24:02g~B*35:31~C*03:04g~DQB1*03:02g~DRB1*04:07g | 0.00110128950165 |
| A*11:01g~B*14:01g~C*08:02g~DQB1*02:01g~DRB1*07:01g | 0.00108452767008 |
| A*02:01g~B*52:01g~C*12:02g~DQB1*06:01g~DRB1*15:02g | 0.00102241255098 |

**Supplementary Table 5.** Linkage disequilibrium for each of the 5 loci.

|  | **HLA-A** | **HLA-B** | **HLA-C** | **HLA-DQB1** | **HLA-DRB1** |
| --- | --- | --- | --- | --- | --- |
| HLA-A | * | + | + | + | + |
| HLA-B | + | * | + | + | + |
| HLA-C | + | + | * | + | + |
| HLA-DQB1 | + | + | + | * | + |
| HLA-DRB1 | + | + | + | + | * |

**Supplementary Table 6.** Allele frequencies for the DRB3, DRB4, and DRB5 loci.

| **HLA-DRB3** | **Frequency** | **HLA-DRB4** | **Frequency** | **HLA-DRB5** | **Frequency** |
| --- | --- | --- | --- | --- | --- |
| 02:02:01G | 0.15574 | 01:01:01G | 0.32070 | 01:01:01G | 0.06967 |
| 01:01:02G | 0.14139 | 01:03:01N | 0.01127 | 01:02:01G | 0.00820 |
| 03:01:01G | 0.06045 |  |  | 02:02:01G | 0.04201 |
| 02:01:01G | 0.00717 |  |  |  |  |

**Supplementary Table 7.** The most frequent A~B~C~DQB1~DRB1 haplotypes in the 8 departments analyzed.

| **Bogotá (n = 981)** | |
| --- | --- |
| **Haplotype** | ***Frequency*** |
| A*24:02g~B*35:43g~C*01:02g~DQB1*03:02g~DRB1*04:07g | 0.0410 |
| A*29:02g~B*44:03g~C*16:01g~DQB1*02:01g~DRB1*07:01g | 0.0224 |
| A*01:01g~B*08:01g~C*07:01g~DQB1*02:01g~DRB1*03:01g | 0.0198 |
| A*33:01g~B*14:02g~C*08:02g~DQB1*05:01g~DRB1*01:02g | 0.0112 |
| A*03:01g~B*07:02g~C*07:02g~DQB1*06:02g~DRB1*15:01g | 0.0110 |
| **Antioquia (n = 163)** | |
| **Haplotype** | ***Frequency*** |
| A*24:02g~B*35:43g~C*01:02g~DQB1*03:02g~DRB1*04:07g | 0.0307 |
| A*02:01g~B*18:01g~C*05:01g~DQB1*02:01g~DRB1*03:01g | 0.0245 |
| A*02:01g~B*44:02g~C*05:01g~DQB1*05:01g~DRB1*01:01g | 0.0184 |
| A*24:02g~B*40:02g~C*03:05g~DQB1*03:02g~DRB1*04:07g | 0.0153 |
| A*03:01g~B*35:01g~C*04:01g~DQB1*05:01g~DRB1*01:01g | 0.0153 |
| **Valle del Cauca (n = 122)** | |
| **Haplotype** | ***Frequency*** |
| A*24:02 g~B*35:43 g~C*01:02 g~DQB1*03:02 g~DRB1*04:07 g | 0.0205 |
| A*02:01 g~B*14:02 g~C*08:02 g~DQB1*05:01 g~DRB1*01:02 g | 0.0165 |
| A*01:01 g~B*08:01 g~C*07:01 g~DQB1*02:01 g~DRB1*03:01 g | 0.0164 |
| A*29:02 g~B*44:03 g~C*16:01 g~DQB1*02:01 g~DRB1*07:01 g | 0.0123 |
| A*29:02 g~B*57:01 g~C*07:01 g~DQB1*05:01 g~DRB1*01:01 g | 0.0123 |
| **Atlántico (n = 115)** | |
| **Haplotype** | ***Frequency*** |
| A*29:02g~B*44:03g~C*16:01g~DQB1*02:01g~DRB1*07:01g | 0.0219 |
| A*01:01g~B*08:01g~C*07:01g~DQB1*02:01g~DRB1*03:01g | 0.0219 |
| A*24:02g~B*35:12g~C*04:01g~DQB1*03:01g~DRB1*16:02g | 0.0175 |
| A*24:02g~B*35:01g~C*04:01g~DQB1*03:01g~DRB1*11:02g | 0.0132 |
| A*03:01g~B*07:02g~C*07:02g~DQB1*06:02g~DRB1*15:01g | 0.0132 |
| **Cundinamarca (n = 70)** | |
| **Haplotype** | ***Frequency*** |
| A*24:02g~B*35:43g~C*01:02g~DQB1*03:02g~DRB1*04:07g | 0.0500 |
| A*29:02g~B*44:03g~C*16:01g~DQB1*02:01g~DRB1*07:01g | 0.0286 |
| A*24:02g~B*35:12g~C*04:01g~DQB1*03:01g~DRB1*16:02g | 0.0214 |
| A*02:01g~B*35:43g~C*01:02g~DQB1*03:02g~DRB1*04:07g | 0.0143 |
| A*02:05g~B*40:02g~C*03:05g~DQB1*04:02g~DRB1*08:02g | 0.0143 |
| **Boyacá (n = 41)** | |
| **Haplotype** | ***Frequency*** |
| A*24:02g~B*35:43g~C*01:02g~DQB1*03:02g~DRB1*04:07g | 0.0976 |
| A*01:01g~B*08:01g~C*07:01g~DQB1*02:01g~DRB1*03:01g | 0.0488 |
| A*02:13~B*51:01g~C*15:02g~DQB1*03:02g~DRB1*04:04g | 0.0244 |
| A*25:01g~B*18:01g~C*12:03g~DQB1*06:02g~DRB1*15:01g | 0.0244 |
| A*29:02g~B*44:03g~C*16:01g~DQB1*02:01g~DRB1*07:01g | 0.0244 |
| **Santander (n = 35)** | |
| **Haplotype** | ***Frequency*** |
| A*24:02g~B*35:43g~C*01:02g~DQB1*02:01g~DRB1*07:01g | 0.0571 |
| A*02:01g~B*07:02g~C*07:02g~DQB1*06:02g~DRB1*15:01g | 0.0286 |
| A*33:01g~B*39:05g~C*07:02g~DQB1*04:02g~DRB1*08:02g | 0.0286 |
| A*24:02g~B*40:02g~C*03:05g~DQB1*03:02g~DRB1*04:07g | 0.0286 |
| A*03:01g~B*38:01g~C*12:03g~DQB1*02:01g~DRB1*03:01g | 0.0286 |
| **Tolima (n = 35)** | |
| **Haplotype** | ***Frequency*** |
| A*24:02g~B*07:02g~C*07:02g~DQB1*06:02g~DRB1*15:01g | 0.0429 |
| A*29:02g~B*44:03g~C*16:01g~DQB1*02:01g~DRB1*07:01g | 0.0286 |
| A*68:01g~B*35:31~C*03:04g~DQB1*03:02g~DRB1*04:07g | 0.0286 |
| A*33:01g~B*14:02g~C*08:02g~DQB1*02:01g~DRB1*03:01g | 0.0286 |
| A*02:01g~B*14:02g~C*08:02g~DQB1*05:01g~DRB1*01:02g | 0.0143 |
